# Graphene Oxide Nanosheets Toxicity in Mice Is Dependent on Protein Corona Composition and Host Immunity

**DOI:** 10.1021/acsnano.4c08561

**Published:** 2024-08-07

**Authors:** Yue-ting Li, Kuo-Ching Mei, Revadee Liam-Or, Julie Tzu-Wen Wang, Farid N. Faruqu, Shengzhang Zhu, Yong-lin Wang, Yuan Lu, Khuloud T. Al-Jamal

**Affiliations:** † School of Cancer & Pharmaceutical Sciences, Faculty of Life Sciences & Medicine, King’s College London, Franklin-Wilkins Building, London SE1 9NH, U.K.; ‡ State Key Laboratory of Functions and Applications of Medicinal Plants, Guizhou Provincial Key Laboratory of Pharmaceutics, Guizhou Medical University, No. 9, Beijing Road, Yunyan District, Guiyang 550004, China; § School of Pharmacy and Pharmaceutical Sciences, State University of New York at Binghamton, 96 Corliss Avenue, Johnson City, New York 13790, United States; ∥ Department of Pharmacology and Pharmacy, Li Ka Shing Faculty of Medicine, The University of Hong Kong, Hong Kong Special Administrative Region, Hong Kong SAR, China; ⊥ Qiannan People’s Hospital, No. 9, Wenfeng Road, Duyun 558000, China

**Keywords:** proteomics, nanotoxicity, biocompatibility, nanomaterials, nanomedicine, immune response, 2D materials

## Abstract

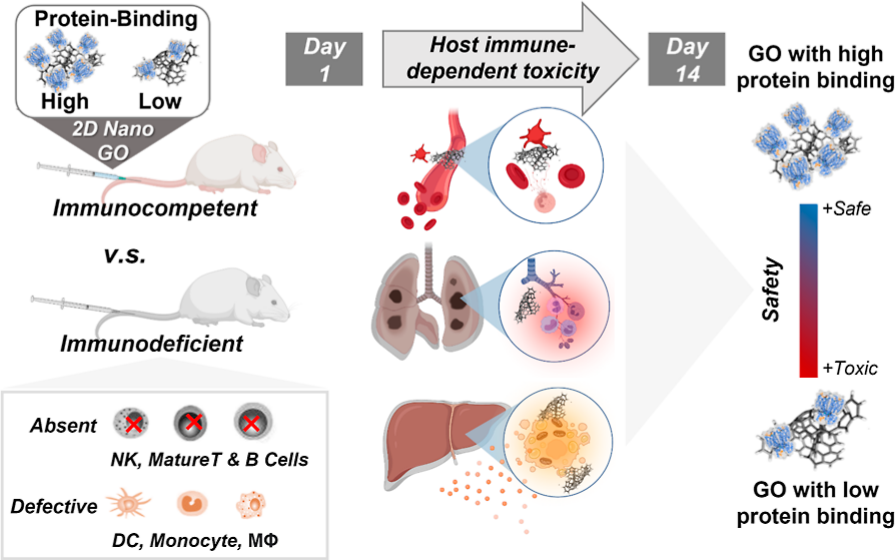

Two-dimension graphene oxide (GO) nanosheets with high and low serum protein binding profiles (high/low hard-bound protein corona/HC_high/low_) are used in this study as model materials and screening tools to investigate the underlying roles of the protein corona on nanomaterial toxicities *in vivo*. We proposed that the *in vivo* biocompatibility/nanotoxicity of GO is protein corona-dependent and host immunity-dependent. The hypothesis was tested by injecting HC_high/low_ GO nanosheets in immunocompetent ICR/CD1 and immunodeficient NOD-*scid II2rγ*^*null*^ mice and performed histopathological and hematological evaluation studies on days 1 and 14 post-injection. HC_low_ GO induced more severe acute lung injury compared to HC_high_ GO in both immunocompetent and immunodeficient mice, with the effect being particularly pronounced in immunocompetent animals. Additionally, HC_low_ GO caused more significant liver injury in both types of mice, with immunodeficient mice being more susceptible to its hepatotoxic effects. Moreover, administration of HC_low_ GO resulted in increased hematological toxicity and elevated levels of serum pro-inflammatory cytokines in immunocompromised and immunocompetent mice, respectively. Correlation studies were conducted to explore the impact of distinct protein corona compositions on resulting toxicities in both immunocompetent and immunodeficient mice. This facilitated the identification of consistent patterns, aligning with those observed *in vitro*, thus indicating a robust *in vitro–in vivo* correlation. This research will advance our comprehension of how hard corona proteins interact with immune cells, leading to toxicity, and will facilitate the development of improved immune-modulating nanomaterials for therapeutic purposes.

## Introduction

Oxidized derivatives of 2D graphene sheets, i.e., graphene oxide (GO), have attracted significant attention for their potential biomedical applications, such as biosensors and delivery carriers of drugs, due to the large specific surface area and versatility of surface functionalization strategies.^1−4^ However, unsatisfactory biocompatibility and adverse effects, such as hemocompatibility,^5^ organ toxicities,^6^ immunotoxicity,^7^ genotoxicity,^8^ and epigenotoxicity^9^ of graphene-based nanomaterials, limit its further development. The protein corona, a critical aspect affecting biocompatibility and nanotoxicity, refers to the formation of a protein shell on the surface of nanoparticles when exposed to biological fluids. GO is no exception, forming various “coronas”, including the hard protein corona (HC), which strongly binds to the nanoparticles’ surface. This HC alters the surface conformation and physicochemical properties of GO, thereby influencing its interactions with biological entities such as cells and tissues, ultimately dictating cellular and tissue responses.^10−12^ Cellular responses to graphene sheets, encompassing toxicity, reactive oxygen species (ROS) production, lipid peroxidation, and inflammation, have been reported to be highly dependent on the HC quantity and composition, as well as the involvement of the immune system.^11−15^ However, the majority of these studies have been conducted *in vitro* due to practical constraints caused during *in vivo* investigations, leaving a significant gap in our understanding of how these responses correlate with *in vivo* conditions. This disparity between *in vitro* and *in vivo* studies hinders the ability to accurately predict the clinical applicability of such nanomaterials.

In previous studies, we synthesized GO and further derivatized it with azide-and-alkyne (Click^2^ GO) functional groups by click chemistry.^16,17^ We found that azide- and alkyne-functionalization lowered protein absorption on GO from 1.4 w/w to 0.8 w/w (protein/GO weight ratios).^18^ The findings from the cellular uptake and cytotoxicity assays conducted *in vitro* indicate that the cytotoxic effects observed were closely linked to alterations in the surface chemistry, particularly influenced by the quantity of protein corona formed.^19^ Reduced binding of serum proteins led to a marked increase in the uptake of GO and subsequent cytotoxicity in phagocytic cells, notably macrophages.^18,19^ These associations were established using a statistical experimental design approach known as Design of Experiments (DoE). Furthermore, the Principal Component Analysis (PCA) was employed to delineate the respective contributions of individual components of the protein corona to these biological responses following GO treatment *in vitro*. Through this process, we could decipher the connection between vitamin D binding protein , inter-alpha-trypsin inhibitor heavy chain H2, beta-2 glycoprotein 1, and POTE Ankyrin domain family member F, and their association with decreased cell viability and increased oxidative stress.

**Figure 1:**
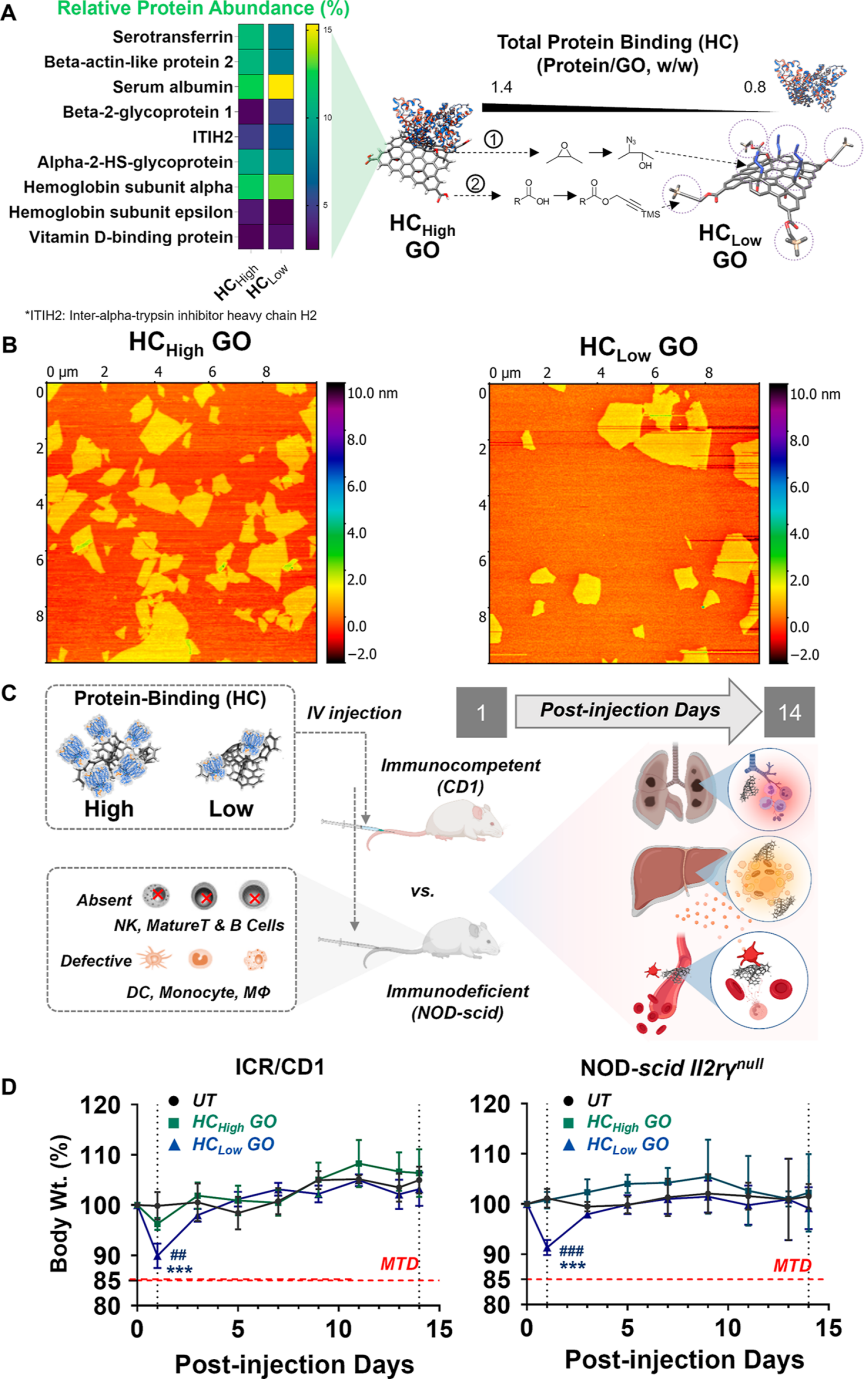
Investigating protein corona-dependent toxicity of GO nanosheets in immunocompetent and immunodeficient mice. (A) Representative illustration of high and low serum protein binding GO previously reported by our group.^18^ Proteins to GO ratios calculated in our previous study were 1.4 and 0.8 w/w for HC_high_ and HC_low_ GO, respectively. Protein corona composition expressed as RPA % came from our previous study, where they were analyzed by LC–MS/MS (Table S1). The nine distinctly different hard corona proteins between the HC_high_ and HC_low_ GO are shown on the left, which was determined by orthogonal partial least-squares discriminant analysis (OPLS-DA). (B) AFM images of HC_high_ and HC_low_ GO are shown. HC_high_ and HC_low_ GO had a mean lateral dimension of 8.9 and 8.0 μm, measured by atomic force microscopy. (C) HC_high_ and HC_low_ GO were injected intravenously into immunocompetent and immunodeficient mice. Histopathological and biochemical analyses were carried out on days 1 and 14 post-injection. (D) HC_low_ GO induced significant but transient bodyweight reduction on day 1 postinjection in immunocompetent and immunodeficient mice, which recovered on day 3. Data expressed as mean values ± standard deviation (SD), *n* = 4. ***p < 0.001 compared to untreated group; ^##^*p* < 0.01, ^###^*p* < 0.001 compared to HC_high_ GO.

This research was conducted to examine the influence of GO chemistry-dependent HC profile on *in vivo* biocompatibility/nanotoxicity and to assess if the outcomes align with the correlational patterns observed in our prior *in vitro* investigation. The overarching goal is to establish a robust correlation approach bridging *in vitro* and *in vivo* contexts. Additionally, the study intends to elucidate the involvement of immune cells in relation to GO and the associated hard protein corona. Building upon our prior *in vitro* investigations, this study focused on evaluating the biocompatibility of chemically functionalized GO and Click^2^ GO variants previously synthesized and characterized. These variants, denoted as high serum protein binding GO (HC_high_ GO) or low serum protein binding GO (HC_low_ GO), were differentiated based on protein-to-graphene weight ratios of 1.4 and 0.8 w/w, as illustrated in Figure 1A (right panel), mean lateral dimensions, and *I*_D_/*I*_G_ ratios (Figure 1B). Notably, these two classes of GOs exhibited significant disparities not only in total HC protein binding but also in the composition of nine serum proteins, namely serotransferrin, beta-actin-like protein 2, serum albumin, beta-2-glycoprotein 1, interalpha-trypsin inhibitor heavy chain H2 (ITIH2), alpha-2-HS-glycoprotein, hemoglobin subunit alpha and subunit epsilon, and vitamin D-binding protein (Figure 1A, left panel). To study the contributory roles of the immune system on *in vivo* toxicity, GOs were intravenously administered to immunocompetent ICR/CD1 and immunodeficient NOD-Prkdc^scid^II2rg^tm1Wjl^/SzJ (*NOD-scid II2rγ*^*null*^) mice. The latter model lacks natural killer (NK) and matured T & B cells. Additionally, it has defective dendritic cells, monocytes, and macrophages.^20^ Histopathological and serum biochemical analyses were carried out on days 1 and 14 post-injection (Figure 1C). Subsequent analysis was performed to correlate the role of the individual corona proteins with *in vivo* toxicities.

## Results and Discussion

It has been reported that the protein corona adsorbed on the surface of nanomaterials governs their-uptake and subsequent polarization/activation in macrophages, which is attributed to cellular/tissue responses, such as ROS production and innate and adaptive immune responses.^11,13,21^ Our earlier *in vitro* study showed that cellular uptake was directly and solely related to the quantity of HC, regardless of protein composition. Meanwhile, nanotoxicity, evaluated through a retrospective design of experiment (DoE), primarily hinges on the uptake, followed by HC composition and exposure dose. Consequently, lower serum protein binding resulted in notably higher uptake of GO and increased cytotoxicity in phagocytic cells, such as macrophages.^18 ,19^ In this study, we sought to elucidate the effects of chemistry-induced changes in the hard protein corona on *in vivo* biocompatibility and nanotoxicity in immunocompetent and immunodeficient mice. Due to difficulties in isolating GO from mice for post-administration proteomics analysis, our investigations relied on *in vitro* data derived from samples incubated with fetal bovine serum (FBS), extrapolated to correlate with *in vivo* outcomes. However, we strengthened our investigations by rigorously correlating *in vitro* protein corona formation with *in vivo* outcomes using diverse statistical approaches.

### GO Induces Organ Coefficient Changes in the Lungs and Liver But Not in the Heart, Spleen, and Kidney

Major organs were harvested on days 1 and 14 after intravenous (i.v.) HC_high/low_ GO injections at maximum tolerating doses. HC_low_ GO induced significant but transient bodyweight reduction at day 1 post-injection in immunocompetent and immunodeficient mice, which recovered on day 3 (Figure 1D). This finding was in line with the superior pro-oxidative potential of HC_low_ GO when compared to HC_high_ GO samples found *in vitro* (Figure S1). Neither organ coefficient nor histological changes were observed in the heart, spleen, and kidney of ICR/CD1 and NOD-*scid II2rγ*^*null*^ mice (Figures S2–S5).

### Immunocompetent Mice Are More Susceptible to Acute Lung Injury Induced by HC_low_ GO

**Figure 2:**
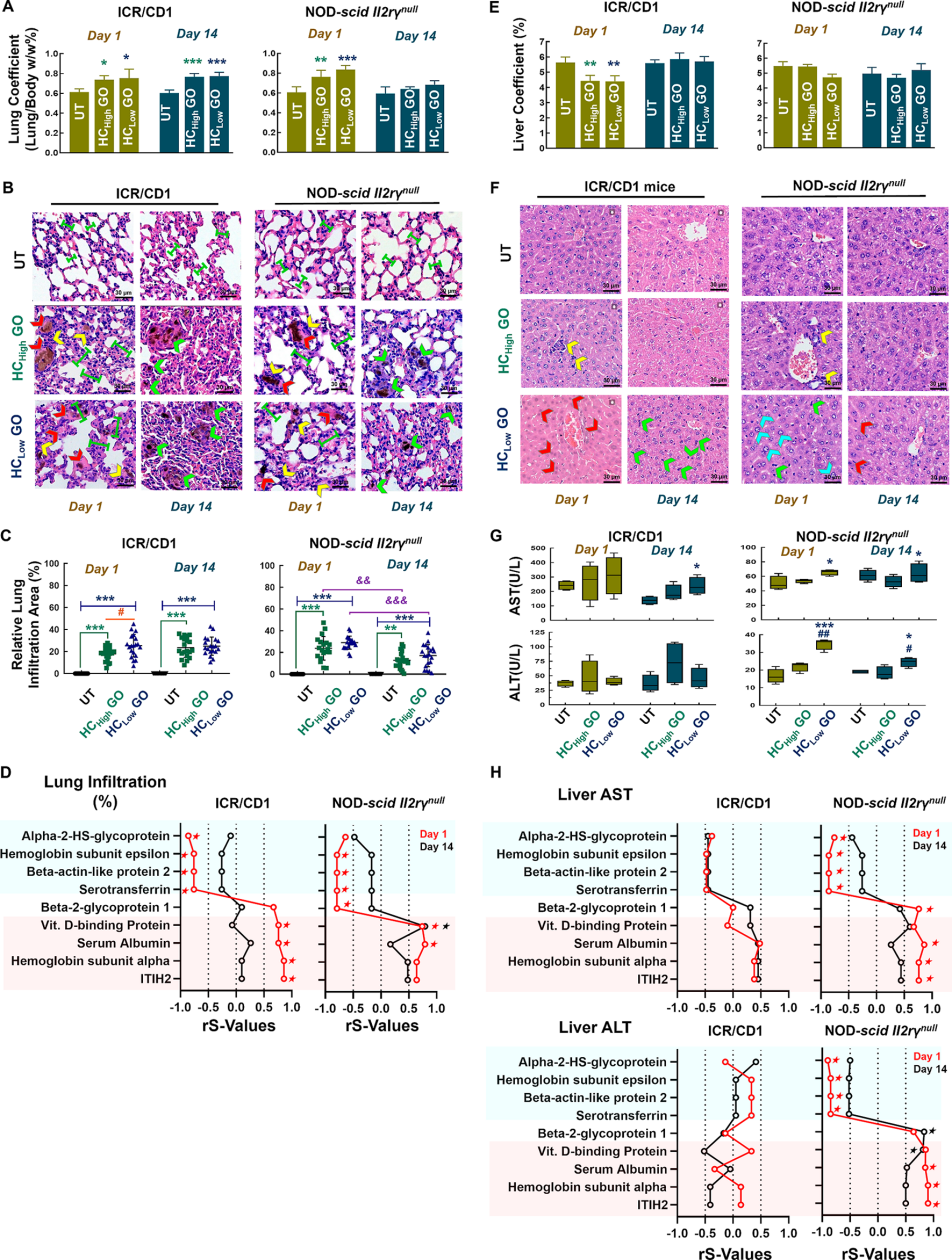
GO induces lung and liver injuries that are protein corona-and-immune system-dependent. (A,E) Lung and liver organ coefficients (lung/bodyweight w/w %) in CD1 and NOD-*scid II2rγ*^*null*^ mice on days 1 and 14 post-injection of HC_high/low_ GO. (B,F) H&E staining of lung and liver sections, respectively. Lung: Alveolar septum thickening (green scale bar), edema (red arrows), neutrophil infiltration (yellow arrows), granuloma formation (green arrows). Liver: neutrophil infiltration (yellow arrows), karyopyknosis and acidophilic degeneration (red arrows), hepatocyte steatosis (green arrows). Ballooning degeneration of hepatocyte and spotty necrosis (blue arrows) (scale bar = 30 μm). (C,G) Relative pulmonary infiltration area (%) of lung and serum AST(ALT) levels in two animal models, respectively. (D,H) Spearman’s correlation analysis was used to evaluate the contribution of protein corona to the observed lung or liver injury, respectively. Proteins with a *p*-value less than 0.05 were significantly correlated to the injury. Data expressed as mean values ± standard deviation (SD), *n* = 4. *p < 0.05, **p < 0.01, ***p < 0.001 compared to the untreated group; ^#^*p* < 0.05, ^##^*p* < 0.01 compared to HC_high_ GO group; ^&&^*p* < 0.01, ^&&&^*p* < 0.001 compared to the same group on day 14; ^★^*p* < 0.05, considered a significant association between protein corona and lung infiltration (%)/elevated level of serum AST (ALT).

Significantly increased lung coefficients were found in ICR/CD1 mice on days 1 and 14 post-injection of HC_high/low_ GO, with a more pronounced increase on day 14. A similar observation was found in NOD-*scid II2rγ*^*null*^ mice, but only on day 1, not on day 14 (Figure 2A). H&E staining of lung sections (Figure 2B) showed alveolar septum thickening (green scale bar), edema (red arrows), and neutrophil infiltration (yellow arrows) (HC_low_ GO, day 1), suggesting acute lung parenchyma injury. Granuloma formation (indicated by green arrows) and diffuse pulmonary fibrosis were observed in ICR/CD1 mice on day 14. The findings suggest persistent lung injury occurred, potentially driven by the disruption of M1/M2 macrophage homeostasis. This disruption appears to be due to GO-induced oxidative stress, which elevates pro-inflammatory factors. Similar phenomena were observed with other graphene materials.^6,22−24^ Both HC_high_ and HC_low_ GO showed more lung infiltration than untreated mice. Lung infiltration persisted until day 14 in immune-competent ICR/CD1 mice but subsided in immune-deficient NOD-*scid II2rγ*^*null*^ mice, consistent with the lung coefficient data (Figure 2C). This also indicates the significant contribution of the innate immune system to lung inflammation as immunodeficient mice lack key innate immune cells,^20^ potentially weakening chronic inflammatory processes. These processes involve local proliferation and polarization of recruited immune cells, ultimately leading to long-term tissue damage.^25,26^

Spearman’s correlation analysis was used to evaluate the contribution of the nine distinctly different hard corona proteins bound on HC_high_ and HC_low_ GO (Figure 1A, left panel), potentially influencing the observed acute lung injury. Proteins with a *p*-value (less than 0.05) were considered to correlate with lung injury (Figure 2D). In immunocompetent ICR/CD1 mice, all corona proteins except beta-2-glycoprotein 1 correlate well with lung infiltration on day 1. Serum albumin, ITIH2, hemoglobin subunit alpha, and vitamin D-binding protein were positively correlated with the observed acute lung injury. In contrast, serotransferrin, beta-actin-like protein 2, alpha-2-HS-glycoprotein, and hemoglobin subunit epsilon showed negative correlations with lung injury. None of the corona proteins correlated with the lung injury on day 14 (Table S4 upper rows). In immunodeficient NOD-*scid II2rγ*^null^ mice on day 1, serum albumin, beta-2-glycoprotein 1, and vitamin D-binding protein correlated positively with lung infiltration, while serotransferrin, beta-actin-like protein 2, and hemoglobin subunit epsilon showed negative correlations. On day 14, only vitamin D-binding protein maintained a positive correlation with lung infiltration (Table S4 lower rows). Proteins positively associated with acute liver injury exhibit a higher relative protein abundance in the HC_low_ GO group compared to the HC_high_ GO group. On the 14th day, minimal protein correlation was observed with lung injury, suggesting that at this stage, pulmonary damage results from the synergistic actions of multiple factors.

### Immunocompromised Mice Are More Susceptible to Acute Liver Injury Induced by HC_low_ GO

**Figure 3:**
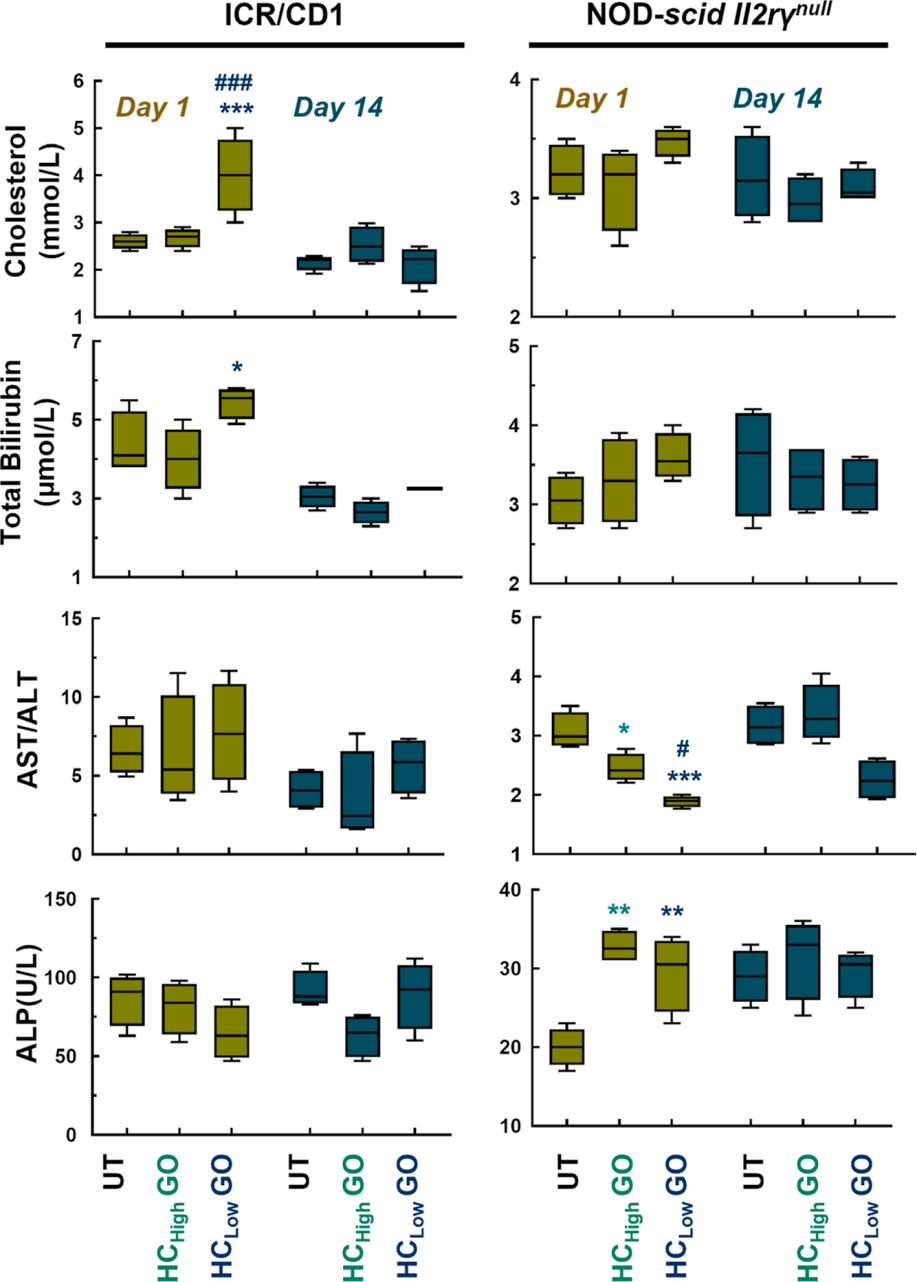
Clinical biochemistry results from ICR/CD1 and NOD-*scid II2rγ*^*null*^ mice treated with HC_high/low_ GO. Serum biochemistry profiles of CD1 and NOD-*scid II2rγ*^*null*^ mice on days 1 and 14 after HC_high/low_ GO exposure. Data are expressed as mean values ± standard deviation (SD), *n* = 4. *p < 0.05, **p < 0.01, ***p < 0.001 compared to the untreated group; ^#^*p* < 0.05, ^###^*p* < 0.001 compared to HC_high_ GO.

Significantly reduced liver coefficients were observed only on day 1 in HC_high_ and HC_low_ GO-treated ICR/CD1 mice (Figure 2E). H&E staining of liver sections (Figure 2F) confirmed predominant neutrophil infiltrates in the portal tract of HC_high_ GO-treated mice on day 1 (yellow arrows). Karyopyknosis and acidophilic degeneration were found in HC_low_ GO-treated ICR/CD1 mice on day 1 (red arrows), progressing to eosinophilic necrosis and hepatocyte steatosis on day 14 as shown by H&E staining (green arrows), indicating that acute liver inflammation in immunocompetent mice caused by HC_low_ GO can advance to chronic immuno-related hepatotoxicity. Ballooning degeneration of hepatocytes and spotty necrosis were found in HC_low_ GO-treated NOD-*scid II2rγ*^*null*^ mice on day 1 (blue arrows). Serum AST(ALT) activities were elevated in HC_low_ GO-treated NOD-*scid II2rγ*^*null*^ mice on both days 1 and 14. Additionally, elevated AST levels were observed in ICR/CD1 mice on day 14, suggesting that liver injury from HC_low_ GO exposure is persistent in immunocompromised mice (Figure 2G). The more pronounced acute liver injury in immunodeficient mice might be due to pro-inflammatory liver sinusoidal endothelial cells (LSECs). Previous studies suggest that LSECs uptake GO nanosheets via scavenger receptors, recruiting leukocytes into the liver.^27^ The balance and retention of immune subsets in the liver determine whether acute injury resolves, persists, or progresses to chronic conditions. Immunodeficiency was reported to render LSECs more susceptible to altering their phenotype toward pro-inflammatory events.^28^ Biochemistry analysis confirmed increased cholesterol and total bilirubin levels in ICR/CD1 mice but decreased levels of serum AST(ALT) and elevated levels of serum ALP in NOD-*scid* mice after 1 day of exposure to HC_low_ GO (Figure 3). These results further confirm the susceptibility of immunodeficient mice to acute liver injury. No other changes were observed in serum albumin, chlorine, urea, sodium, calcium, potassium, inorganic phosphorus, and creatinine in any treatment group (Figure S6–S7).

Spearman’s correlation analysis showed a correlation between nearly all protein corona and serum AST(ALT) activities in immunodeficient NOD-*scid II2rγ*^*null*^ mice on day 1 (Figure 2H). Serum albumin, ITIH2, beta-2-glycoprotein 1, and hemoglobin subunit alpha positively correlated with the level of serum AST(ALT) levels, while serotransferrin, beta-actin-like protein 2, alpha-2-HS-glycoprotein, and hemoglobin subunit epsilon negatively correlated to serum AST(ALT) values (Figure 2H, Table S5 lower rows). Beta-2-glycoprotein 1 and vitamin D-binding protein correlated with ALT levels on day 14 (Figure 2H, Table S6 lower rows). However, no significant correlations were observed between corona proteins and serum AST(ALT) levels in ICR/CD1 mice (Tables S5 and S6 upper rows). The above findings suggest that the immune system plays a crucial role in mediating acute liver injury induced by protein corona. The correlation analysis findings align with those observed in lung injury, indicating a positive association between proteins exhibiting relatively high abundance in HC_low_ GO and the occurrence of acute liver injury. These *in vivo* results appear to be in concordance with our previous *in vitro* data. In our earlier work, we used principal component analysis (PCA) to develop a highly predictive model that explains *in vitro* biointeractions.^18^ PCA condenses data by creating fewer artificial variables called principal components (PCs), which are linear combinations of the original variables (proteins on graphene).^18^ The first principal component (PC-1) captures the most explained variance, while the second principal component (PC-2) captures the second most variance, reflecting the main data trends along these coordinates. Initially, the distinction between HC_high_ GO and HC_low_ GO is evident along the PC-1 axis. We subsequently correlated this PCA outcome with the biological process of cellular uptake by J774.A1. Our findings revealed an inverse correlation: a higher quantity of HC is associated with a decrease in cellular uptake and lower PC-1 scores. In contrast, PC-2 demonstrates a robust correlation with cytotoxicity, thereby indicating its suitability as a reliable measure. The individual contribution of each protein to the principal component can be effectively understood through the PC score table and loading plot. Enrichment of beta-actin-like protein 2, alpha-2-HS-glycoprotein, serotransferrin and serum albumin primarily distinguishes the uptake capacity between HC_high_ GO and HC_low_ GO on PC-1, where a more positive PC-1 indicates enhanced cellular uptake. For the PC-2 axis, vitamin D-binding protein, ITIH2, and beta-2-glycoprotein 1 are responsible for cytotoxicity, with a more negative PC-2 score indicating reduced cellular viability. Cellular uptake is predominantly HC-quantity-dependent, while cytotoxicity is collectively influenced by HC quantity, composition, and graphene exposure dose.

Our *in vivo* results highlight that GO may impact cellular responses through a combination of corona-related factors, both qualitatively and quantitatively. As previously described, we employed a range of statistical tools to unravel these intricate mechanisms underlying GO’s effects and correlate with our previous *in vitro* findings. The evident synergistic effects of both quantity and composition of HC on macrophage uptake and immune response were demonstrated, as observed in lung and liver injury. Spearman’s correlation analysis identifies key proteins contributing to these outcomes, with certain proteins demonstrating concordance with *in vitro* toxicity data, which indicated a sufficiently reliable correlation between protein corona study performed *in vitro* and *in vivo*. For instance, the increased presence of serotransferrin, beta-actin-like protein 2, and alpha-2-HS-glycoprotein (all having negative PC-1 scores and almost neutral–positive PC-2 scores, as noted in our prior study) on HC_high_ GO exhibited negative correlations with lung injury in this study. Conversely, HC_low_ GO, enriched with proteins possessing a strongly positive PC-1 score (serum albumin) and a markedly negative PC-2 score (ITIH2 and vitamin D-binding protein), manifested positive correlations with the observed lung accumulation and acute lung injury (characterized by alveolar septum thickening and neutrophil infiltration observed on day 1 after HC_high/low_ GO exposure). Our finding is relevant to the existing literature describing the functional properties of these proteins. For example, vitamin D-binding protein scavenges G-actin released from damaged lung cells, exacerbating neutrophilic inflammation.^29,30^ Beta-2- glycoprotein 1, involved in innate immunity, activates macrophages to trigger the adaptive immune response.^31,32^ The enrichment of these proteins on HC_low_ GO may potentially over activate the inflammation cascade.

Regarding acute liver injury, the effects were clearly observable in mice with compromised immune function. There was a clear association: the proteins with highly positive PC-1 scores like serum albumin and highly negative PC2 scores, ITIH2 and beta-2-glycoprotein 1 (also enriched on HC_low_ GO), showed a positive correlation with serum AST(ALT) levels. On the contrary, akin to our observations in the lungs, serotransferrin, beta-actin-like protein 2, and alpha-2-HS-glycoprotein (enriched on HC_high_ GO) exhibited an inverse correlation with serum AST(ALT) values.

### HC_low_ GO Induces Hematological Toxicity in Mice

**Figure 4:**
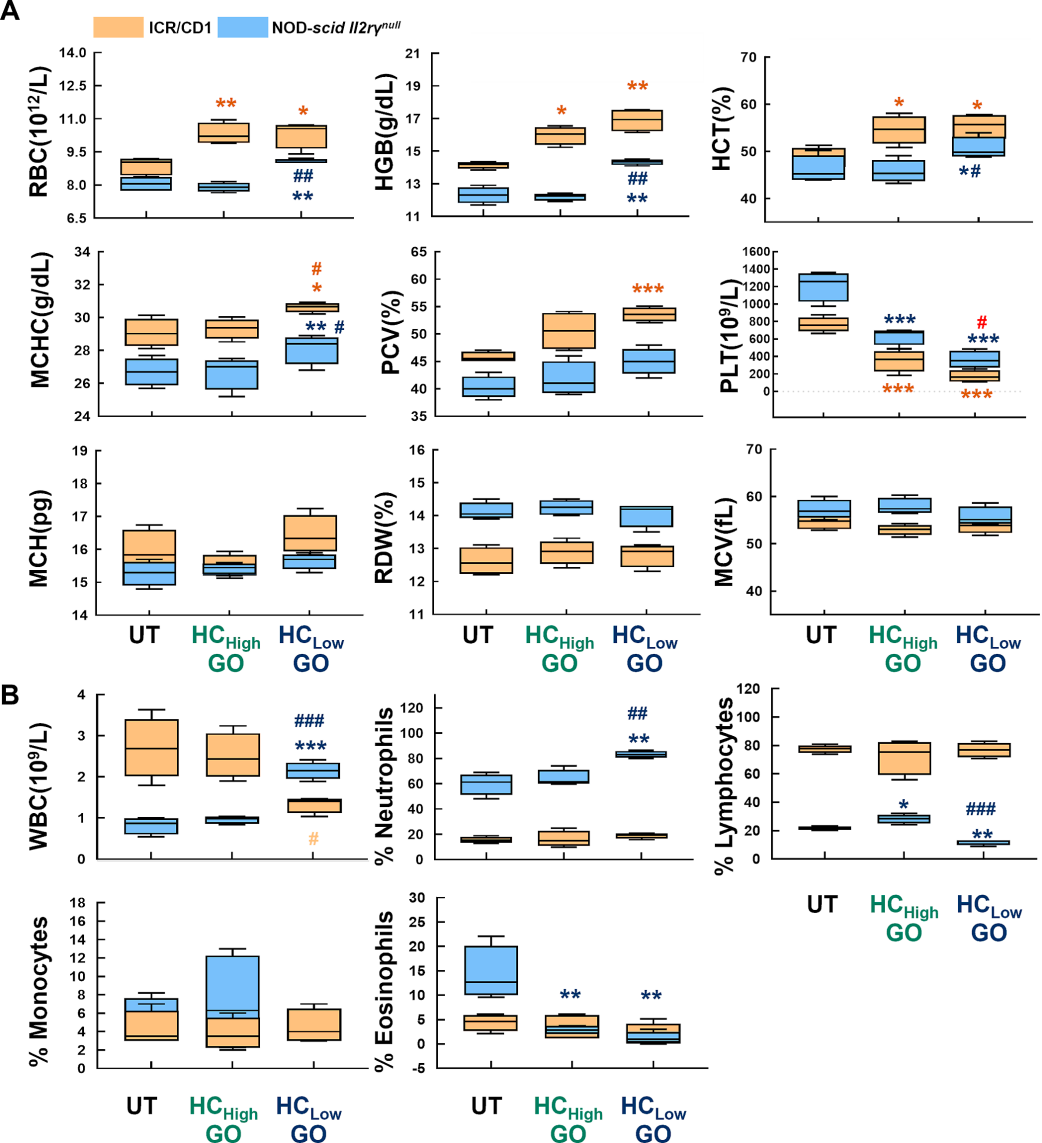
HC_low_ GO induces more severe hematological toxicity than HC_high_ GO. (A) RBC, HGB counts, HCT, MCHC, PCV values, and PLT and (B) WBC and monocyte counts for HC_low_ GO and HC_high_ GO treated ICR/CD1 and NOD-*scid II2rγ*^*null*^ mice. Data are expressed as mean values ± standard deviation (SD), *n* = 4. *p < 0.05, **p < 0.01, ***p < 0.001 compared to untreated group; ^#^*p* < 0.05, ^##^*p* < 0.01, ^###^*p* < 0.001 compared to HC_high_ GO group.

Increased red blood cells (RBC), hemoglobin (HGB) level, hematocrit (HCT) content, mean cell hemoglobin concentration (MCHC), and packed cell volume (PCV) counts were observed in HC_low_ GO-treated ICR/CD1mice and NOD-*scid II2rγ*^*null*^ mice on day 1 (Figure 4A). Unlike HC_low_ GO, HC_high_ GO only induced increased RBC, HGB level, and HCT content in ICR/CD1 mice. No changes in mean corpuscular hemoglobin (MCH), red cell distribution width (RDW), and mean corpuscular volume (MCV) were observed. The administration of HC_low_ GO resulted in decreased platelet (PLT) counts for two animal types. Additionally, HC_low_ GO altered total WBC counts in both mice strains (Figure 4B). NOD-*scid II2rγ*^*null*^ mice showed increased WBC counts (↑ neutrophils, ↓ lymphocytes, ↓ eosinophils), while ICR/CD1 mice displayed reduced total WBC counts, not its subpopulations, on day 1 after receiving HC_low_ GO.

Spearman’s correlation analysis demonstrated that at least one of the nine corona proteins correlated with changes in PLT, MCHC (majorly), MCH, and WBC counts in ICR/CD1 mice (Table S7). In contrast, NOD-*scid II2rγ*^*null*^ mice exhibited more pronounced and diverse associations between the corona proteins and alterations in RBC, HGB, PLT, MCHC, HCT, WBC, neutrophils (%), and lymphocytes (%) (Table S8). Increased RBC, HGB level, and HCT content were observed in HC_low_ GO-treated ICR/CD1 mice and NOD-scid *II2rγ*^*nul*^ mice on day 1. However, it was observed that there was no significant correlation between the levels of RBC, HGB, and HCT content. Through analysis of Kendall’s Coefficient of Concordance (W), a significant correlation was observed between lung injury and RBC, HGB, HCT, MCHC, MCH, and PCV values in both ICR/CD1 mice (coefficient = 0.997, *p*-value <0.001) and NOD-*scid II2rγ*^*null*^ mice (coefficient = 0.959, *p*-value <0.001) (Table S9). This indicates that changes in indicators related to the body’s oxygen transport function, such as erythrocytes, are positively associated with acute lung injury.

Recent studies reported that NP internalization by macrophages through serum albumin binding to scavenger receptors (SRs) plays a key role in macrophage polarization into M1 pro-inflammatory phenotype, initiating the inflammatory cascade and producing reactive oxygen species (ROS).^33−35^ Notably, the toxicity and pro-inflammatory characteristics of HC_low_ GO, influenced by their unique albumin-enriched corona profile, align with their pro-oxidant nature as determined by ROS, GSH, and SOD assays (Figure S1). Prooxidant exposure is also known to induce hematological changes.^36^ Therefore, the increase in RBC, HGB, HCT, MCHC, MCH, and PCV levels following exposure to prooxidant GO (HC_low_ GO) indicates its potency as an inducer of acute lung injury. This mirrors acute lung injury/acute respiratory distress syndrome, characterized by heightened hemolysis and decreased blood oxygen levels due to compromised gas exchange in lung capillaries, leading to elevated HGB and compensatory erythrocyte production.^37,38^ Furthermore, in response to inflammation, coagulation can be activated, leading to decreased platelet count. Our statistical analysis revealed a strong association between this phenomenon and corona components, specifically beta-2-glycoprotein 1and vitamin D-binding protein are known to inhibit platelet activation and aggregation through various mechanisms.^29,39,40^ Hence, besides the heightened inflammatory potential of HC_low_ GO, their higher enrichment of vitamin D-binding protein and beta-2-glycoprotein 1 in the corona, obtained from blood, might reduce serum levels of these proteins, diminishing their biological functions and increasing clotting tendency.^41−43^

### HC_low_ GO Induces Release of Pro-Inflammatory Cytokines and Chemokines

**Figure 5:**
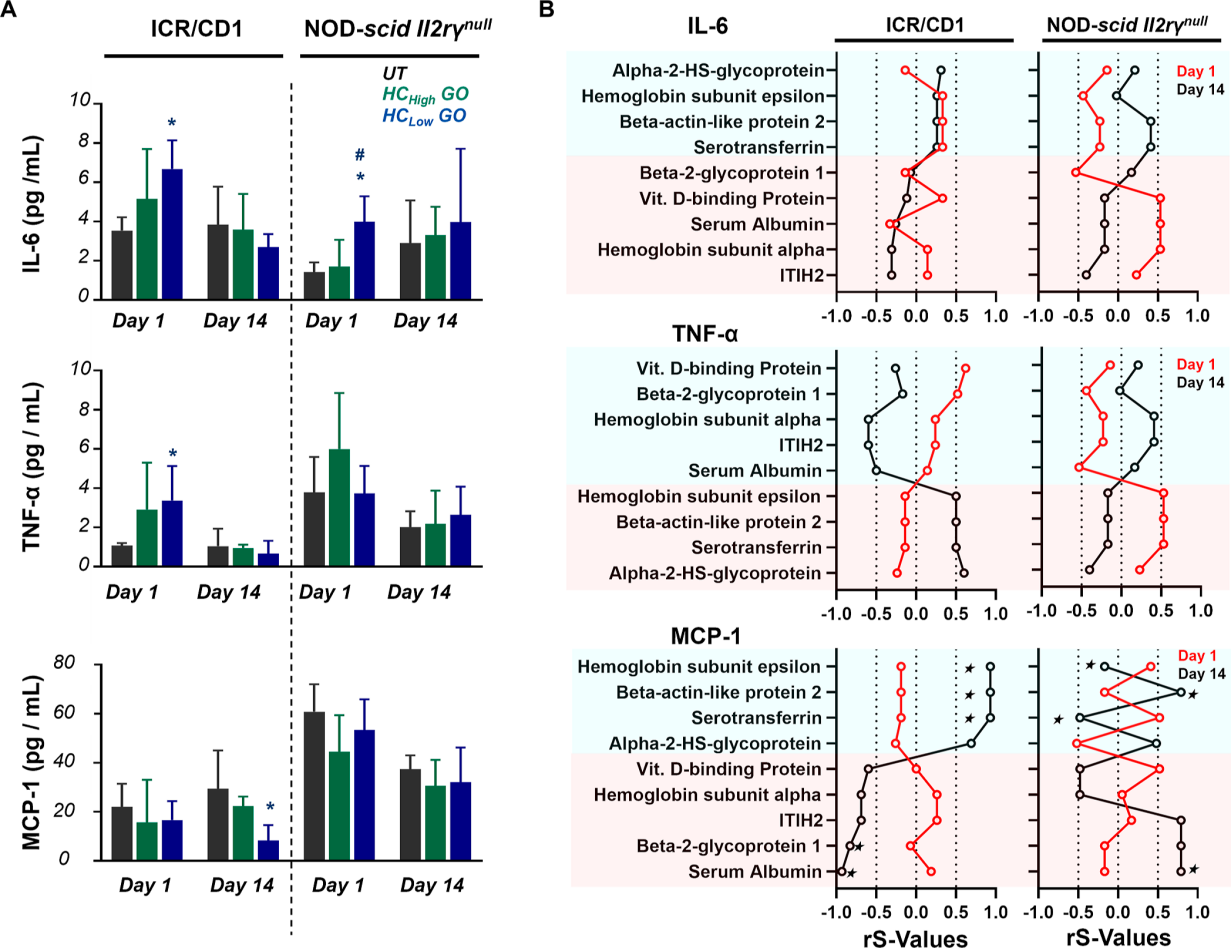
HC_low_ GO induces pro-inflammatory cytokines and chemokines. (A) Pro-inflammatory cytokines IL-6, TNF-alpha, and chemokines MCP-1 in serum quantified by cytometric bead array (CBA) flex sets. (B) Spearman’s correlation analysis was used to evaluate the contribution of protein corona to pro-inflammatory cytokines and chemokines responses. Proteins with a *p*-value less than 0.05 were considered to be significantly correlated to cytokine and chemokine secretion. Data are expressed as mean values ± standard deviation (SD), *n* = 4. *p < 0.05, compared to the untreated group; ^#^*p* < 0.05 compared to the HC_high_ GO group. ^★^*p* < 0.05, considered a significant association between protein corona and MCP-1.

The levels of serum pro-inflammatory cytokines IL-6 and TNF-α increased 1 day after the treatment with HC_low_ GO in ICR/CD1 mice, and chemokine MCP-1 decreased on day 14 (Figure 5A). The levels of serum IL-6 increased in NOD-*scid II2rγ*^*null*^ mice 1 day after exposure to HC_low_ GO. No changes were observed in the HC_high_ GO treatment group. Spearman’s correlation analysis (Figure 5B) showed that in CD1 mice, serotransferrin, beta-actin-like protein 2, and hemoglobin subunit epsilon correlated positively with MCP-1 reduction on day 14. Conversely, serum albumin and beta-2-glycoprotein 1 correlated negatively with MCP-1 reduction (Figure 5B, Table S10 bottom panel). In NOD-*scid II2rγ*^*null*^ mice, serotransferrin, beta-actin-like protein 2, serum albumin, and hemoglobin subunit epsilon correlated positively with MCP-1 reduction on day 14.

No apparent correlation was found between corona proteins and changes in serum IL-6 (Table S11) or TNF-α (Table S12) in both CD1 and NOD-*scid II2rγ*^*null*^ mice, which suggest that the elevated levels of serum IL-6 or TNF-α are not directly attributed to proteins. According to Kendall’s Coefficient of Concordance (W), a significant correlation was observed between pro-inflammatory cytokines and lung/liver injuries (Table S13). Additionally, elevated white blood cell (WBC) counts were significantly correlated with both lung and liver injuries (Table S14) in ICR/CD1 and NOD-*scid II2rγ*^*null*^ mice, as well as with pro-inflammatory cytokines (Table S15).

Two-dimensional materials, including GO, can trigger inflammation through various mechanisms, often tied to specific factors such as the presence of distinct functional groups (e.g., PEGylation, which facilitates physical interaction with cell membrane and induces cytokine secretion^44^), surface exposure to recipient cells, and the prevalence of particular types of corona proteins (e.g., apoA-I, which enhances interactions with scavenger receptor B1 on macrophages,^45,46^ or immunoglobulin, which can provoke a pro-inflammatory response^47^). However, our previous statistical analysis revealed that nanotoxicity, as assessed through DoE, primarily hinges on nanomaterial uptake, predominantly mediated by HC quantity. This suggests that HC quantity serves as the primary trigger for the inflammatory cascade. The lower adsorption of the protein corona may leave the GO surface with “pro-inflammatory” chemical groups more exposed, potentially leading to enhanced direct interaction with receptors and subsequent inflammation. This is consistent with the *in vitro* studies conducted by Ge *et al.* and Chong *et al.*,^48,49^ which suggest that the protein corona mitigates the pro-inflammatory effects and cytotoxicity of GO by limiting its interaction with the cell membrane. The surface chemistry properties of carbon nanomaterials, including GO,^50,51^ have also previously been identified as key physicochemical features capable of inducing oxidative stress through the generation of ROS, thus triggering inflammation and inducing toxicity. For instance, it was found that a high level of hydroxyl-functionalization (O and H) in GO is an important determinant of acute inflammation in graphene materials.^51^ Additionally, distinct toxicity can also be attributed to the hydrophobicity and surface oxidation status of GO.^52^ GO with hydrophobic modification has been shown to induce ROS generation mainly due to strong hydrophobic interactions with the cell membrane, which inhibits the uptake of essential nutrients and proteins into cells.^53−55^ Therefore, the presence of hydrophobic functional groups, such as azide and alkyne, on HC_low_ GO could potentially induce inflammation *via* this mechanism. However, the physicochemical nature of GO can be altered upon contact with the biological environment, leading to the formation of a new bioidentity. This phenomenon occurs before GO reaches the recipient cells. Hence, in this study, we considered GO and its adsorbed corona as a unified camouflage system, and the biological effects mediated by GO we observed here are potential synergistic contributions of the protein corona and exposure to specific GO surface chemistry. It is also worth noting that the lateral size of GO and the route of administration can affect protein corona formation and toxicity. Our study specifically examined the intravenous administration of micrometre-scale GO sheets, which are known to provoke a stronger pulmonary pro-inflammatory response than nanometre-sized GO.^23,56−58^ Earlier studies have documented that exposure to nanosized GO is typically well-tolerated, with either transient inflammatory reactions or the absence of adverse effects noted in both rodent models and, more recently, in human subjects.^23,59,60^

As earlier mentioned, the *in vitro* to *in vivo* correlation established in this study is likely to be a function of the surface chemistry of GO, which potentially has a huge contribution to HC-mediated biological interactions *in vitro* and *in vivo*. According to our previous investigation,^18^ the HC quantity is largely dependent on the surface functional groups and steric hindrance, i.e., azide and alkyne attachments onto GO limit the access of proteins and water molecules to the oxygen derivatives on the GO planar surface. This finding is consistent with previous research,^61−63^ which has demonstrated that variation in surface chemistry and hydrophobicity plays a significant role in driving protein adhesion and influencing protein adsorption promotion. Therefore, the surface chemistry factor can exert a similar influence on the quantity of protein formed on GOs in vivo as observed *in vitro*, irrespective of serum protein source.

The study by Townson *et al.*(64) provided evidence supporting the hypothesis that the surface chemistry of nanoparticles (NPs) has a significant impact on both the quantity of HC formed in the presence of FBS and their cellular uptake *in vitro*. This correlation aligns with their findings on *in vivo* cellular interactions investigated across different species environments. In Marques *et al.*’s study,^65^ the *in vitro* single protein-NP studies were comparable to *in vivo* protein corona data, demonstrating the successful translation of direct results from *in vitro* studies into *in vivo* investigations. In our prior research, we aimed to understand HC-mediated cellular behavior using a well-established model of mouse-derived macrophages (J774.A1). This model is potentially translatable to macrophages’ behavior in the lungs and liver of a mouse model. While variations in serum protein composition between FBS and mouse serum are conceivable, potentially resulting in differences in the HC profile, our results, despite utilizing the data obtained from FBS instead of mouse serum, consistently revealed patterns of key proteins influencing cellular uptake and cytotoxicity across various *in vivo* settings, encompassing the lungs, liver, and serum

In our current study, we emphasize the importance of using pre-formed serum protein corona, created with FBS, to establish a connection between *in vitro* behaviors and *in vivo* responses. This assertion is grounded in our prior research and further supported by the *in vitro* experiments conducted in this study. Specifically, our experiments with J774.A1 cells, focusing on uptake (as detailed in the previous study) and cytotoxicity (GSH, SOD, and modified LDH assays performed in this current work), reinforce this approach. These cells were cultured under pre-defined serum protein conditions and recognized as the gold standard supplement for mammalian cell culture. Given this consistency, it is logical to continue using pre-formed serum protein corona to explore the protein corona formation pattern on GO instead of mouse-derived serum. This choice allows us to establish a statistically robust correlation model between the protein corona profile and *in vitro* readouts, which has been demonstrated in the current study to be extrapolatable to the *in vivo* context. We recognize the potential advantages of using mouse serum to improve the translatability of findings from *in vitro* to *in vivo* experiments, particularly in mouse models. However, the introduction of mouse serum requires additional optimization steps in cell culture and *in vitro* experiments to ensure protocol suitability and proper cell function. This concern should be taken into consideration in the future when establishing the correlation model between *in vitro* and *in vivo* settings.

Taken together, it is evident that these hard protein corona-mediated effects were observed *in vivo* and exhibited a statistically significant association with our *in vitro* findings through the application of Principle Component Analysis, Design of Experiment, Spearman’s rank correlation analysis, and Kendall’s coefficient of concordance. Our study focuses on the stable, strongly bound hard protein corona and its effects on GO during biological exposure. However, the potential role of the soft protein corona, with its dynamic and exchangeable nature in response to environmental changes, remains challenging but significant and requires further investigation.

## Conclusion

In conclusion, our study showed that the surface chemistry-driven alterations in hard protein corona resulted in characteristic *in vivo* toxicity profiles, which were shown to be immune-dependent. HC_low_ GO induced intensified acute lung injury relative to HC_high_ GO in immunocompetent and immunodeficient mice. Macrophage-led immune responses favored chronic lung immune toxicity, while immune deficiency correlated with potentiated liver immune toxicity, applicable to the nanomaterial tested. This also highlights the importance of tailored protein corona incorporation in GO development to enhance characteristics conducive to *in vivo* delivery. Despite previous negative associations with diverse nanoparticles, specific proteins adsorbed on GO can mitigate toxicity in systemic delivery. Our study also identifies functional groups to avoid when formulating GO to minimize undesired *in vivo* behavior, informing future designs of 2D nanomaterials for drug delivery and immunotherapeutic applications. It has also proven that nanomedicine safety can be highly dependent on the immune competency status of the animal employed in preclinical studies. Finally, although utilizing macrophage depletion approaches have been shown to enhance the delivery efficacy of nanomedicines effectively, our study suggests that safety considerations should be carefully considered when utilizing such approaches in future studies.

## Materials and Methods

### Preparation of GOs with Different Serum Protein Binding Ability

GOs used in the current study belong to a joint investigation of our previously published work involving the synthesis and functionalization of GO. The unmodified GO was synthesized using Mei’s modified Hummers’ method^17^ and then functionalized by introducing azide and alkynes. The resulting GO and azide/alkyne-functionalized GOs had a mean lateral dimension of 8.9 and 8.0 μm and *I*_D_/*I*_G_ ratios of 1.25 and 1.36, measured by atomic force microscopy and Raman spectroscopy, respectively (Figure 1B).^16,17,66,67^ The total protein binding (HC quantity per mg of graphene materials, protein: GO) was obtained by measuring proteins detached by sodium dodecyl sulfate (SDS), as reported in our previous study^18^ (Figure 1A). Detached protein corona was analyzed using LC–MS/MS as previously described.^18^ Unmodified GO showed higher protein binding with a protein-to-GO ratio of 1.44:1 w/w. Azide/alkyne-modified GO showed a significantly reduced serum protein binding and a protein-to-GO ratio of 0.80:1 w/w.^18^ The same batch of GO was then used for the present study and hereafter named as HC_high_ GO (unmodified, high protein binding) and HC_low_ GO (azide/alkyne-modified, low protein binding).^17^ Hard protein corona (HC) compositions are expressed as relative protein abundance (RPA %) for HC_high_ GO and HC_low_ GO (Table S1).^18^

For animal experiments, the same batch of GO samples was prepared as PBS dispersions at a concentration of 0.5 mg/mL. These samples were intravenously injected into the animals without preformed serum protein corona. This approach was chosen to correlate our *in vitro* data with the pure effect of protein corona formation *in vivo*, thereby eliminating the dynamic interactions and effects between the preformed corona and the corona acquired in the bloodstream.

### Animal Models

Male immunocompetent ICR/CD1 and immunodeficient NOD-Prkdc^scid^II2rg^tm1Wjl^/SzJ (NOD*-scid II2rγ*^*null*^) were selected for the study. NOD-scid (non-obese diabetic/severe combined immunodeficiency) mice are deficient in innate immunity and almost absent in adaptive immunity (Table S2).^68^ All animal experiments were performed in compliance with the UK Animals (Scientific Procedures) Act 1986 and the UK Home Office Code of Practice for the Housing and Care of Animals Used in Scientific Procedures (Home Office 1989). *In vivo* experimentation adhered to the project license approved by the King’s College London animal welfare and ethical review body (AWERB) and UK Home Office (PBE6EB195). ICR/CD1 and NOD-*scid II2rγ*^*null*^ mice (6–8 weeks old) were purchased from Harlan (London, UK) and Charles River (London, UK), respectively. All mice were housed in polycarbonate ventilated cages with free access to autoclaved food and acidified autoclaved water.

### Toxicity Studies at the Maximum Tolerated Dose

Studies were performed in compliance with the general principles of the International Conference on Harmonization (ICH) guideline M3(R2), where one of the approaches in general toxicity studies recommended in non-clinical studies is the extended single dose toxicity designed to evaluate hematology, clinical chemistry, necropsy, and histopathology data after a single administration, with further evaluations conducted 2 weeks later to assess delayed toxicity and recovery. The suggested design for rodents is to assess these end points 1 day and 14 day post-dosing. Single-injection Maximum Tolerated Dose (MTD) was identified via dose-escalation studies through intravenous (i.v.) injection of HC_high/low_ GO at 0.85, 1.7, 3.4, and 6.8 mg/kg, respectively (*n* = 3) in both ICR/CD1 and NOD-*scid II2rγ*^*null*^ mice. Bodyweight changes and mortality were recorded up to 14 days after administration. The MTD was estimated based on the highest 100% surviving dose with no more than 15% weight loss.^69^ The identified MTD was used for toxicity studies (Table S3). For the extended single-dose toxicity study, ICR/CD1 and NOD-*scid II2rγ*^*null*^ mice were randomly assigned to three groups: (i) vehicle (PBS), (ii) HC_high_ GO, and (iii) HC_low_ GO. Mice were i.v. administered with HC_high/low_ GO at MTD and sacrificed 1 or 14 days after injection (*n* = 12/group). Toxicity evaluations included changes in body weight and organ coefficient (organ/total body w/w %), levels of pro-inflammatory blood cytokines, and alterations in organ histopathology and clinical biochemistry (*n* = 4/group/time point, 1 and 14 days after injection). The additional study included blood compatibility evaluation (*n* = 4 per treatment, 1 day after injection).

### Blood Compatibility Evaluations

One day after HC_high/low_ GO injection in mice, whole blood samples were collected *via* cardio puncture. Some collected blood was mixed with ethylenediaminetetraacetic acid dipotassium (K2 EDTA, as an anticoagulant) to prepare air-dried blood smears for hematological profiling. The remaining blood was allowed to clot for 1 h at room temperature and then centrifuged at 1300 g (Eppendorf 5810R, rotor: F45-30-11) for 8 min at 4 °C to obtain serum for biochemical analysis (sodium, potassium, chloride, calcium, inorganic phosphorus, urea, creatinine, cholesterol, and liver function tests, for example, the total bilirubin, albumin, alanine transaminase (ALT), aspartate transaminase (AST), and alkaline phosphatase (ALP). Haematological, biochemical, and histological analyses were performed by the Royal Veterinary College (London, UK)

Pro-inflammatory cytokines and chemokines were quantified using BD Cytometric Bead Array (CBA) Flex Sets (BD Biosciences). Cytokine standards were prepared by serial dilutions according to the manufacturer’s instructions. Detection limits were 1.4, 2.7, and 2.8 pg/mL for Interleukin 6 (IL-6), monocyte chemoattractant protein-1 (MCP-1, a.k.a. CCL2), and tumor necrosis factor-α (TNF-α), respectively. Beads were run on an LSRFortessa flow cytometer (BD Biosciences). Data were analyzed using the FCAP Array software (BD Biosciences).

### Histopathological Examination

The heart, liver, spleen, lung, and kidney were harvested and fixed in 10% neutral buffered formalin. Samples were then wax-embedded and sectioned for hematoxylin and eosin (H&E) staining according to standard histological protocols at the Royal Veterinary College (London, UK). Microscopy examinations were performed to examine typical lung inflammation characteristics, such as alveolar septum thickening, edema, neutrophil infiltration, formation of granulomas, and diffuse pulmonary fibrosis. Areas of alveolar septum thickening, edema, neutrophil infiltration, and formation of granulomas were manually segmented using Image-Pro Plus software (Media Cybernetics). Relative pulmonary infiltration (%) of these histological features was calculated by comparing the area of histological features/total area of the lung section (*x* 100%) and normalized to the % infiltration in comparison with that of the untreated group (*n* = 20 randomly chosen microscopic fields/group).^70^

### Comparative Analysis of Protein Corona Abundance Variation

In our current study, we built upon previous quantitative protein profiles^18^ to conduct a more in-depth statistical comparison analysis. Distinctly different hard corona proteins between HC_high_ and HC_low_ GO were determined by supervised orthogonal partial least-squares discriminant analysis (OPLS-DA). OPLS-DA was performed using SIMCA-P + V13 software (Umetrics). Different hard corona proteins were determined by variables important in projection (VIP) > 1 and an absolute value of correlation coefficient (|*P*(corr)| > 0.6) (Table S1).

### Statistical Analysis

Quantitative data are presented as mean ± standard deviation (*n* = 4). Statistical significance was determined by one-way ANOVA (post hoc = Tukey or Dunnett’s T3 tests) using the IBM SPSS version.^67^ A p-value of less than 0.05 was considered statistically significant. In addition, spearman’s rank correlation analysis^71,72^ was performed to define the correlation between relative protein abundance (RPA %) of different HC proteins and toxic biological responses. A two-tailed p-value of less than 0.05 indicates the association. rS-values (Spearman’s rank correlation coefficients) close to −1 or +1 represent stronger relationships than values closer to zero. rS-sign indicates positive and negative correlations for positive and negative signs, respectively.

Finally, Kendall’s coefficient of concordance (Kendall’s W)^72^ was used to measure the concordance of damage degree of lung injury with oxygen transport functions, including red blood cell (RBC), hemoglobin (HGB), hematocrit (HCT), mean cell hemoglobin concentration (MCHC), mean cell hemoglobin (MCH) and packed cell volume (PCV). Kendall’s W was also used to evaluate the concordance of cytokine (and chemokines) levels with lung injury, liver injury, and white blood cell (WBC) counts. Additionally, concordance of WBC counts with lung and liver injuries was estimated in Kendall’s W.

## Supporting Information Available

Figures S1–S8 detailing other organs coefficients and H&E Staining, clinical biochemistry; Additional tables (Table S1–Table S15) on immunodeficiency of NOD-scid II2rγnull mice, mortality, VIP and P(corr) of 9 Distinctly Different Hard Corona Proteins, correlation analysis between notable proteins and in vivo nanotoxicity; Pro-oxidative potential of HCHigh/Low GO (PDF)
