# Supplementary material for: Graphene Oxide Nanosheets Toxicity in Mice Is Dependent on Protein Corona Composition and Host Immunity
Source: ACS Nano. 2024 Aug 7;18(33):22572–85. doi: 10.1021/acsnano.4c08561 (PMC11342366; doi:10.1021/acsnano.4c08561)
Supplement: Supplementary file 1 — nn4c08561_si_001.pdf [file nn4c08561_si_001.pdf]

Supporting Information

Graphene Oxide Nanosheets Toxicity in Mice is Dependent on Protein Corona Composition and Host Immunity

Yue-ting Li,[a, b, #] Kuo-Ching Mei,[a, c, #] Revadee Liam-Or, [a d, #] Julie Tzu-Wen Wang,[a] Farid N. Faruqu,[a] Shengzhang Zhu,[e] Yong-lin Wang,[b] Yuan Lu,[a,b] Khuloud T. Al-Jamal[a,d*]

[a] School of Cancer & Pharmaceutical Sciences, Faculty of Life Sciences & Medicine, King's College London, Franklin-Wilkins Building, London SE1 9NH, UK

[b] State Key Laboratory of Functions and Applications of Medicinal Plants, Guizhou Provincial Key Laboratory of Pharmaceutics, Guizhou Medical University, No. 9, Beijing Road, Yunyan District, Guiyang, 550004, China

[c] School of Pharmacy and Pharmaceutical Sciences, State University of New York at Binghamton, 96 Corliss Ave, Johnson City, NY 13790, USA

[d] Department of Pharmacology and Pharmacy, Li Ka Shing Faculty of Medicine, The University of Hong Kong, Hong Kong Special Administrative Region, China

[e] Qiannan People's Hospital, No. 9, Wenfeng Road, Duyun, 558000, China

[#] Contributed equally to the work

*Corresponding email: khuloud.al-jamal@kcl.ac.uk

Supporting Materials and Methods

Materials

DCFDA Cellular Reactive Oxygen Species (ROS) Detection Assay Kit (cat: ab113851) were purchased from Abcam (UK), Glutathione (GSH) Assay Kit (cat: CS0260) was purchased from Sigma (UK), Superoxide Dismutase (SOD) determination kit (cat: 19160) were purchased from Sigma (UK). Cytotox 96® Non-Radioactive Cytotoxicity Assay Kit (cat: G1780) was purchased from TECHNICAL BULLETIN (UK). Penicillin Streptomycin (P/S, 10,000 U/mL, 10,000 µg/mL) (cat: 15140122), Advanced Roswell Park Memorial Institute (Adv. RPMI) medium (cat: 12633012) and trypsin EDTA (0.25%, phenol red, cat: 25200056) were obtained from Thermo Fisher Scientific (UK). J774A.1 (BALB/cN mouse macrophage, ATCC®TIB-67™) was obtained from ATCC®.

Methods

Assessing the pro-oxidative potential of $HC_{High/Low}$ GO, using reactive oxygen species (ROS), glutathione (GSH), and superoxide dismutase (SOD) assay

ROS assay

DCFDA Cellular ROS Detection Assay Kit was used for ROS assay (following the manufacturer's instructions). J774 cells were cultured in Adv. RPMI (both with 1% P/S, 1% GlutaMAX™, and 10% NCS). Cells were maintained in a T75 cell culture flask until 80% confluency (37˚C, 5% CO_2) before seeding into 12-well plates. J774 cells (2.5×10^4/well) were seeded onto 96-well plates and allowed to be set for 24 h. Freshly prepared graphene samples (HC_{High} GO and HC_{Low} GO) at 10, 50, and 100 µg/mL prepared with complete media without phenol were used and incubated with the cells for 24h. Two types of blank wells were prepared: Blank 1 (media only) and Blank 2 (with no cells but with graphene samples at the same concentration). Positive control wells (containing cells but none of the graphene samples) were arranged simultaneously. Tert-Butyl Hydrogen Peroxide (TBHP) Solution (500 µM) was spiked into positive control wells by adding 11 µL per well 4 hours before completion of treatment. The sample wells, positive control wells, and blank wells were added 100 µL of 2X

DCFDA dilution per well, then continually incubated for the 45min. Fluorescence intensity was detected by fluorescence spectroscopy with maximum excitation and emission spectra of 485nm and 535nm, respectively, in a FLUOstar Omega microplate reader (BMG Labtech, Germany). Folds increased in ROS production was calculated using the following equation: $(F_{test}–F_{blank2})/(F_{control}–F_{blank1})$.

GSH assay

J774 cells were cultured in Adv. RPMI (both with 1% P/S, 1% GlutaMAX™, and 10% NCS). Cells were maintained in a T75 cell culture flask until 80% confluency (37˚C, 5% CO_2) before seeding into 6-well plates. J774 cells were seeded in different 6-well plates (3 x 10^5/well) 24 h before the incubation with graphene samples. Freshly prepared graphene samples (HC_{High} GO and HC_{Low} GO) at 10, 50, and 100 μg/mL were used and incubated with the cells for 24 h (1 mL/well). H_2O_2 was used as a positive control at a concentration of 2.43 mmol/L. After 24 h incubation, ice-chilled PBS gently rinsed the remaining still viable cells. Nonadherent dead cells were aspirated with PBS (1 mL/well). Rinsed viable cells were trypsinised by 300 μL 0.05% trypsin-EDTA at 37˚C for 3 min before adding the serum-containing cell culture media to stop the trypsinisation (500 μL/well). Cell suspensions were transferred into 2 mL centrifuge tubes and centrifuged at 4,000 rpm (Beckman Coulter Allegra X-22R Benchtop Centrifuge, rotor: F2402H) for 10 min at 4˚C. The supernatant was removed.

The washed cell pellets were then resuspended in 100 uL 5% 5-Sulfosalicylic Acid (SSA) and frozen-thawed twice to obtain the cell lyse. The cell lysates were centrifuged at 14,000 rpm (Eppendorf 5810R, rotor: F45-30-11) for 10 min at 4˚C to obtain the supernatant for GSH activity evaluation using Glutathione Assay Kit in 96-well plates.

In brief, the two reagent blank samples comprised 5% SSA (10 μL) and a working mixture (150 μL). Glutathione Standard Solutions were prepared: various dilutions of 10 μL samples of the prepared Glutathione Standard Solutions+150 μL working mixture. The unknown samples were constituted of the unknown sample in duplicate (X μL), 5% SSA (up to 10 μL sample),

and a working Mixture (150 µL). After Incubating for 5 minutes at room temperature, these three samples were added 50µL NADPH (0.16 mg/mL) and then mixed. The UV absorbance of each well was measured at 412 nm using a microplate reader (FLUOstar Omega, BMG Labtech) with Omega software (v2.1). The standard curve was determined by the values of the Glutathione Standard Solutions, then the $\triangle A_{412}$/min equivalent to 1 nmole of reduced glutathione per well was calculated. The nmoles of GSH in the unknown sample were calculated using the following equation:

The nmoles of GSH in the unknown sample=($\triangle A_{412}$/minn(sample)$\times$ *dil*)/ ($\triangle A_{412}$/minn(nmoles) $\times$vol) where *dil* represents the dilution factor of the original sample and vol represents the volume of sample in the reaction in mL. The % GSH depletion was calculated by normalizing to the untreated control cells.

SOD assay

Mei's method was used for the SOD assay with some adjustments.[9] J774 cells were cultured in Adv. RPMI (both with 1% P/S, 1% GlutaMAX™, and 10% NCS). Cells were maintained in T75 cell culture flasks until 80% confluency (37˚C, 5% CO_2) before seeding into 12-well plates. J774 cells were seeded in different 12-well plates (8 x 10^4/well) 24 h before the incubation with graphene samples. Freshly prepared graphene samples HC_{High} GO and HC_{Low} GO) at 10, 50, and 100 µg/mL were used and incubated with the cells for 24 h (1 mL/well). H_2O_2 was used as a positive control at a concentration of 2.43 mmol/L. After 24 h incubation, ice-chilled PBS gently rinsed the remaining still viable cells. Nonadherent dead cells were aspirated with PBS (1 mL/well). Rinsed viable cells were trypsinised by 300 µL 0.05% trypsin-EDTA at 37˚C for 3 min before adding the serum-containing cell culture media to stop the trypsinisation (500 µL/well). Cell suspensions were transferred into 2 mL centrifuge tubes and centrifuged at 4,000 rpm (Beckman Coulter Allegra X-22R Benchtop Centrifuge, rotor: F2402H) for 10 min at 4˚C. The supernatant was discarded. The washing step was repeated twice (1.5 mL PBS/tube). The washed cell pellets were resuspended in 200 µL PBS and frozen-thawed twice

to obtain the cell lyse. The cell lysates were centrifuged at 14,000 rpm (Eppendorf 5810R, rotor: F45-30-11) for 15 min at 4˚C to obtain the supernatant for SOD activity evaluation using the SOD determination kit in 96-well plates.

In brief, three types of blank control samples were prepared: Blank 1 (20 μL double distilled H_2O + 20μL of enzyme working solution + 200 μL water-soluble tetrazolium/WST working solution), Blank 2 (20 μL cell lyse + 20 μL dilution buffer + 200 μL WST working solution), and Blank 3 (20 μL double distilled H_2O + 20 μL dilution buffer + 200 μL WST working solution). The samples were constituted of cell lyse (20 μL), WST working solution (200 μL), and enzyme working solution (20 μL). The samples and the blank controls were mixed thoroughly within the 96-well plate and then incubated at 37 °C for 20 min. The UV absorbance of each well was measured at 440 nm using a microplate reader (FLUOstar Omega, BMG Labtech) with Omega software (v2.1). The SOD activity was calculated using the following equation:

$SOD\ Activity = \{[(A_{440\ Blank\ 1} - Abs_{440\ Blank\ 3}) - (A_{440\ Sample} - A_{40\ Blank\ 2})]/(A_{440\ Blank\ 1} - A_{450\ Blank\ 3})\} \times 100\%$. The relative SOD activity was calculated by normalizing the untreated control cells.

Assessment of cellular viability by a modified lactate dehydrogenase (mLDH) assay

The modified lactate dehydrogenase (mLDH) assay was used, *i.e.,* instead of measuring the LDH released, the LDH remained within the cell and was analysed to minimise the interference from the graphene[10,16]. J774 cells (1 x 10^4/well) were seeded onto 96-well plates and allowed to be set for 24 h. Freshly prepared graphene samples (HC_{High} GO and HC_{Low} GO) at 10, 50, and 100 μg/mL were used and incubated with the cells for 24 h. DMSO (10% in cell culture media) was used as a positive control for the assay, as it causes cytotoxicity by interfering with membrane permeability. After 24 h incubation, cell culture media was carefully and slowly removed using multichannel pipettes. The remaining cells were lysed in phenol-red and serum-free Adv. RPMI containing 0.9% Triton X-100 at 37˚C for 1h (100 μL/well). The cell lysates were centrifuged at 4000 rpm (Eppendorf 5810 R) at 4˚C for 1 h to precipitate the graphene within the cell lyse. The supernatant was carefully recovered (to avoid graphene

samples precipitate), and the CytoTox96® LDH assay kit was used for cytotoxicity assay, following the manufacturer's instructions. Absorbance at 490 nm was measured in the FLUO star Omega microplate reader (BMG Labtech, Germany). Cell viability was calculated as the percentage of control untreated cells using the following equation: {(A490 of treated cells - A490 of negative control)/(A490 of untreated cells-A490 of negative control)} × 100. Negative control: phenol-free Adv. RPMI containing 0.9% Triton X-100.

Supporting Results

The pro-oxidative potential of $HC_{High/Low}$ GO

HC_{Low} GO, with strongly pro-oxidative potential, produced more ROS than HC_{High} GO. Additionally, HC_{Low} GO was found to deplete cellular GSH by 50.74%, 86.50%, and 94.46%, while HC_{High} GO achieved 29.75%, 50.95%, and 46.41% GSH depletion in J774 cells at the concentrations of 10, 50, and 100 μg/mL, respectively. Superoxide dismutase (SOD) activity in J774 cells decreased sequentially, similar to the cell viability trend, *i.e.*, HC_{High} GO> HC_{Low} GO (Figure **S1**).

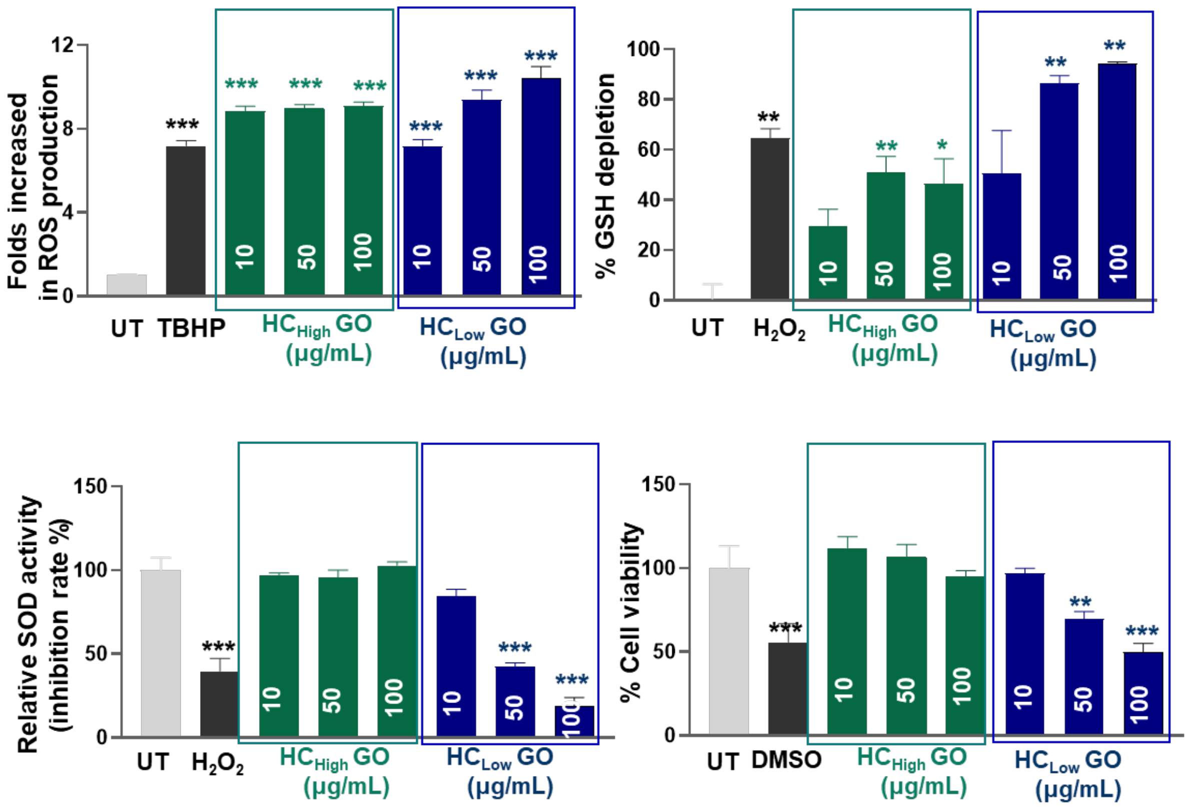

Figure S1. Assessing the pro-oxidative potential and cytotoxicity of HC$_{High/Low}$ GO. Pro-oxidative potential assessment of HC$_{High/Low}$ GO in J774 cells by ROS, GSH, and SOD assay. Cytotoxicity of HC$_{High/Low}$ GO was assessed by the modified lactate dehydrogenase (mLDH) assay. After exposure to HC$_{High/Low}$ GO for 24h, more ROS was produced, and GSH depletion was observed. Lower relative SOD activities and cell viability were observed only in HC$_{Low}$ GO-treated J774 cells, while no other changes were observed in HC$_{Hight}$ GO-treated J774 cells. Data expressed as mean values ± standard deviation (SD), n = 4. * p<0.05, ** p<0.01, *** p<0.001 compared to the untreated group.

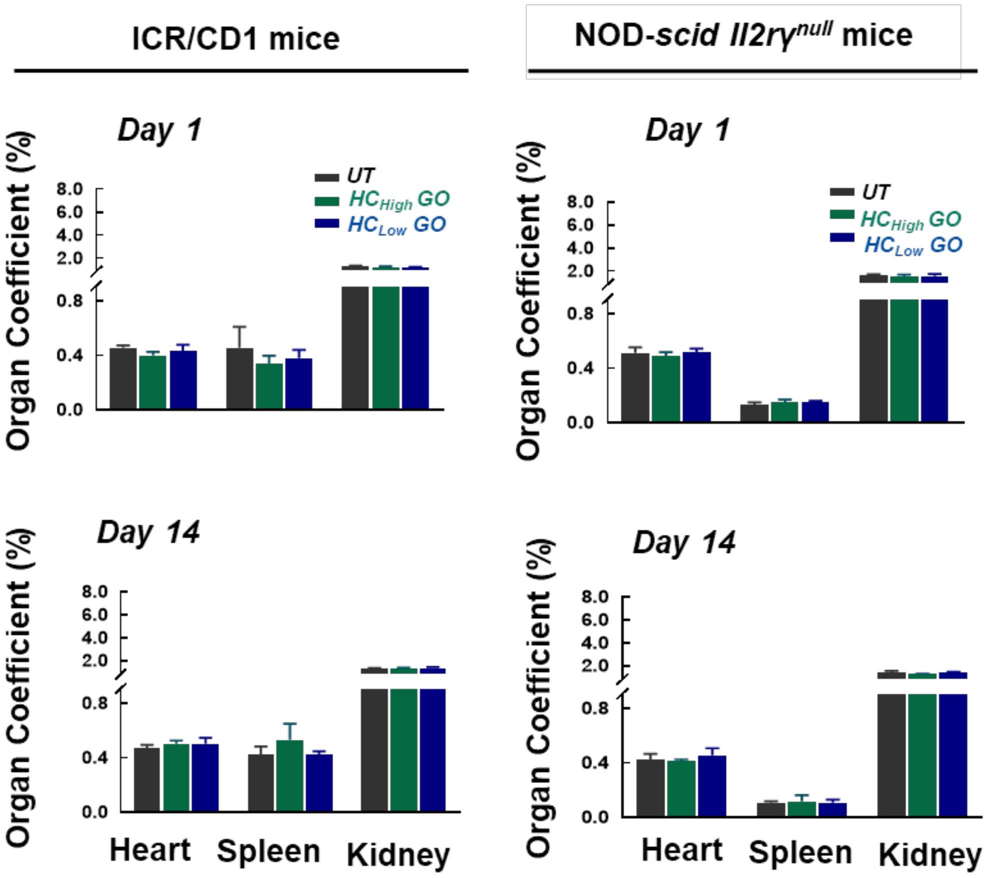

Figure S2. Organ Coefficients of Heart, Spleen, and Kidney in ICR/CD1 and NOD-*scid* *Il2ry*^{null} mice on Days 1 and 14 after IV Injections of HC$_{High/Low}$ GO.

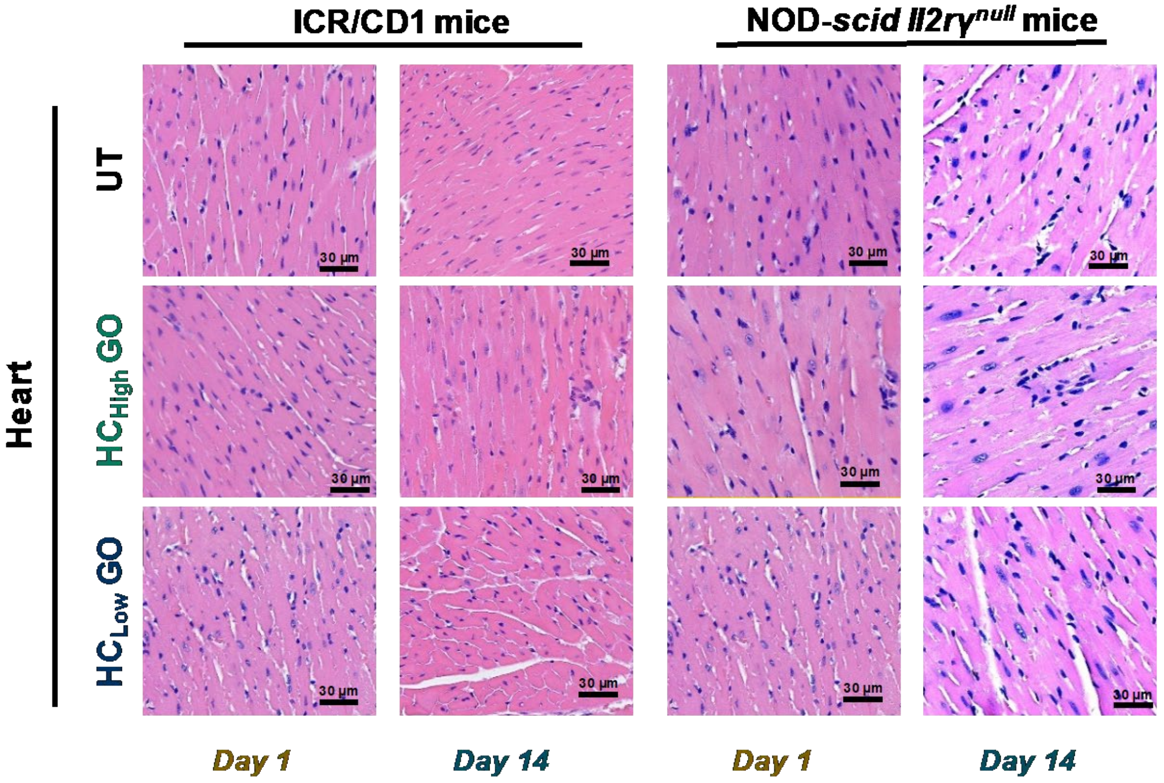

Figure S3. H&E Staining of Heart. Tissue sections in ICR/CD1 and NOD-*scid Il2rγ*^{null} mice were obtained for histopathological analysis using *H&E* staining. (Scale bar = 30 µm)

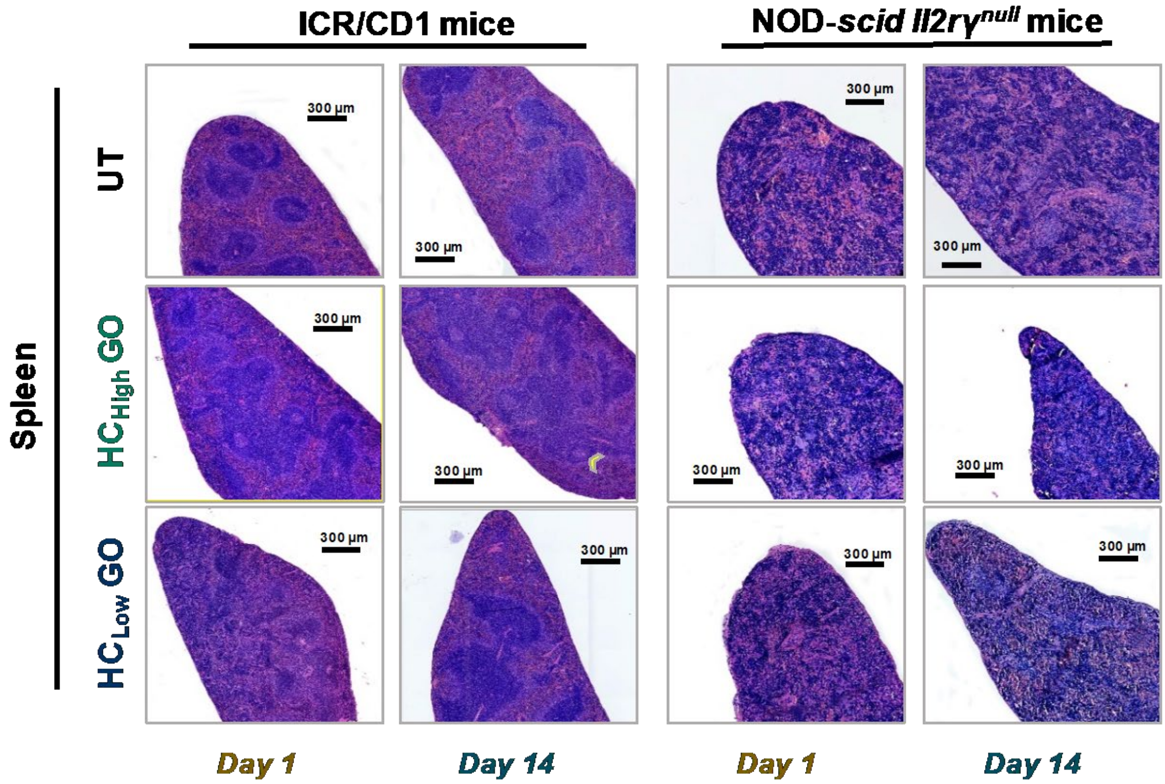

Figure S4. H&E Staining of Spleen. Tissue sections in ICR/CD1 and NOD-*scid Il2rγ*^{null} mice were obtained for histopathological analysis using *H&E* staining. (Scale bar = 300 µm).

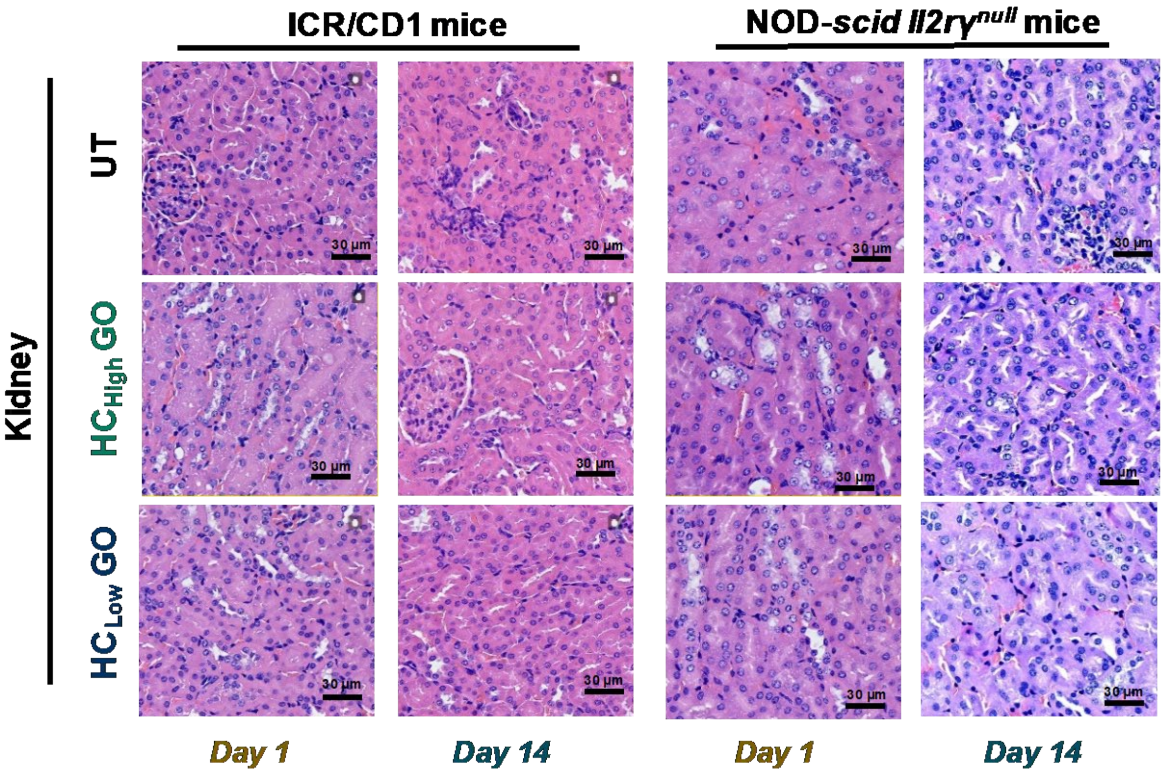

Figure S5. H&E Staining of Kidney. Tissue sections in ICR/CD1 and NOD-*scid Il2rγ*^{null} mice were obtained for histopathological analysis using *H&E* staining. (Scale bar = 30µm)

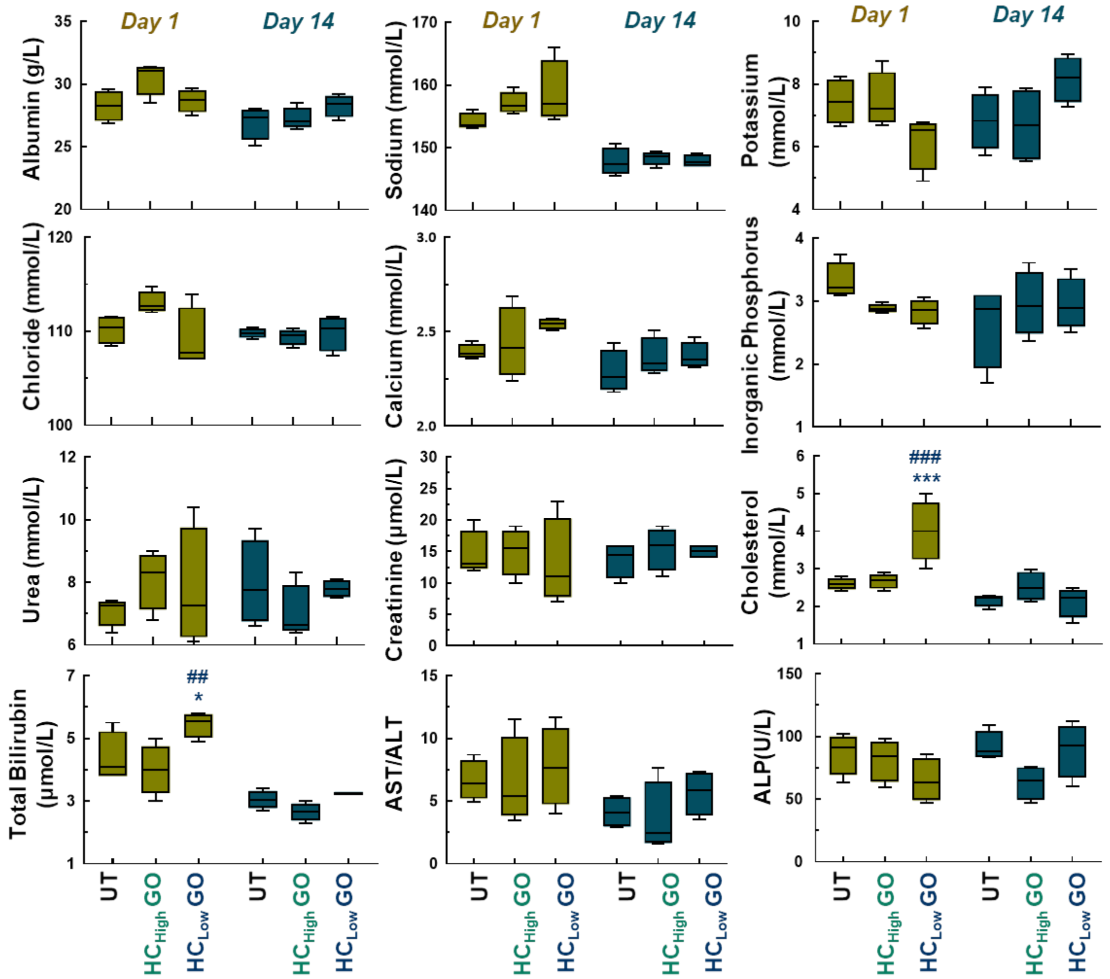

Figure S6. Clinical Biochemistry Results from ICR/CD1 Mice Treated with $HC_{High/Low}$ GO. Healthy ICR/CD1 mice were dosed intravenously with $HC_{High/Low}$ GO at the dose of MTD. Half of the animals in each group were assessed on the day following dosing, and the remaining were assessed on day 14. Serum biochemistry profiles of ICR/CD1 mice exposed to $HC_{High/Low}$ GO. were analysed at 1- and 14-day post-expose, respectively. HC_{Low} GO ICR/CD1 mice detected higher cholesterol and total bilirubin at 1-day post-expose. Data expressed as mean values ± standard deviation (SD), *n* = 4. $^{*}p < 0.05$, $^{***}p < 0.001$ compared to untreated group; $^{\#\#}p < 0.01$, $^{\#\#\#}p < 0.001$ compared to HC_{High} GO.

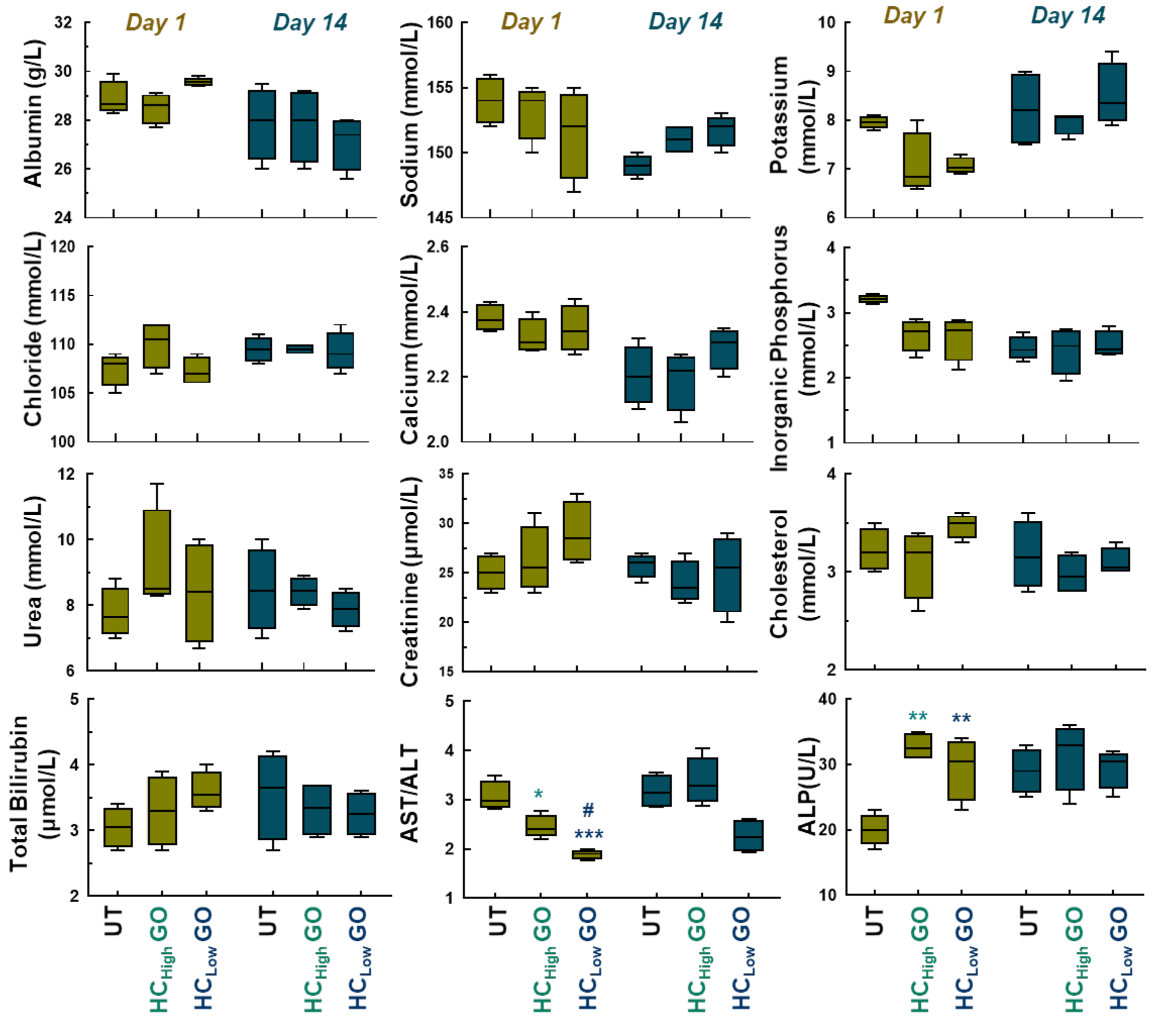

Figure S7. Clinical Biochemistry Results from NOD-*scid Il2rγ*^{null} Mice Treated with HC$_{\text{High/Low}}$ GO. Healthy NOD-*scid Il2rγ*^{null} mice were single-dose treated intravenously with HC$_{\text{high/low}}$ GO at the dose of MTD. Half of the animals in each group were assessed on the day following dosing, and the remaining animals were assessed on day 14. Serum biochemistry profiles of NOD-*scid Il2rγ*^{null} mice exposed to HC$_{\text{High/Low}}$ GO were analysed at 1- and 14-day post-exposure. A decrease of AST/ALT and higher ALP were observed in HC$_{\text{Low}}$ GO-treated mice at 1-day post-exposure. Data are expressed as mean values ± standard deviation (SD), $n = 4$. *$p < 0.05$, **$p < 0.01$, ***$p < 0.001$ compared to the untreated group; $^{\#}p < 0.05$ compared to HC$_{\text{High}}$ GO.

Table S1. The relative protein abundance (RPA%), ***VIP*** **and** ***P(corr)*** **of 9 Distinctly Different Hard Corona Proteins.**

Protein	RPA% [a]		Different Hard Corona Proteins	
	HC_{High} GO	HC_{Low} GO	VIP	*P(corr)*
Serotransferrin	11.3	8.1	2.588	-0.933
Beta-actin-like protein 2	11.1	8.2	2.567	-0.999
Serum albumin	12.7	15.8	2.566	0.999
Beta-2-glycoprotein 1	2.9	5.2	2.274	1.000
ITIH2	4.9	7	1.952	0.873
Alpha-2-HS-glycoprotein	10.2	8.7	1.690	-0.825
Hemoglobin subunit alpha	12.7	13.7	1.588	0.866
Hemoglobin subunit epsilon	3.5	2.6	1.475	-0.983
Vitamin D-binding protein	2.7	3.3	1.125	0.977

a citing the data source are from previously published data[18]

Abbreviations: *ITIH2: inter-alpha-trypsin inhibitor heavy chain H2 ; VIP: variable important in projection; P(corr):value of correlation coefficient*

Table S2. Immunodeficiency of NOD-*scid Il2rγ*^{null} mice

Immune cells	Immune properties	Immune response involved	Function
Natural killer cells	Absent	Innate immunity	Exert their cytolytic effects; Produce a variety of cytokines and chemokines regulate the activities of other immune cells .
Dendritic cells	Defective	Innate immunity and adaptive immunity	Phagocytosis (with monocytes and macrophages, comprise the mononuclear phagocyte system); Initiate T cell immune response.
Macrophages	Defective	Innate and adaptive immunity	Phagocytic activity; Secretion of pro-inflammatory cytokines factors;
Mature B cells	Absent	adaptive immunity	To secrete the specific antibody to bind an antigen;
Mature T cells	Absent	adaptive immunity	Control antibody responses, activate innate immune cells, and lyse target cells.

Table S3. Mortality was observed in ICR/CD1 and NOD-*scid Il2rγ*^{null} Mice at Varying Doses of HC_{High} GO Or HC_{Low} GO During MTD Studies

Host Animal	HC Quantity on GO	Dose (mg/kg)	Weight change % (Day 7)	Instant Death (<5 min)/Total
Immune Competent (ICR/CD1)	Untreated	PBS	0.79	0/3
	High	0.85	3.03	0/3
		1.7	3.20	0/3
		3.4	2.88	0/3
		6.8	0.35	2/3
	Low	0.85	3.28	0/3
		1.7	0.87	0/3
		3.4	-1.23	0/3
		6.8	-	3/3
Immune Compromised (NOD-*scid Il2rγ*^{null})	Untreated	PBS	2.52	0/3
	High	0.85	3.12	0/3
		1.7	3.6	0/3
		3.4	-	2/3
		6.8	-	3/3
	Low	0.85	0.77	0/3
		1.7	1.61	0/3
		3.4	-	3/3
		6.8	-	3/3

Table S4. Correlation Analysis Between Protein Corona and Relative Lung Infiltration (%)

		1*		2		3		4		5		6		7		8		9	
		rS[#]	*P*	*rS*	*P*	*rS*	*P*	*rS*	*P*	*rS*	*P*	*rS*	*P*	*rS*	*P*	*rS*	*P*	*rS*	*P*
ICR/CD1 mice	Day 1	-0.76	0.03	-0.76	0.03	0.76	0.03	0.67	0.07	0.86	0.01	-0.86	0.01	0.86	0.01	-0.76	0.03	0.76	0.03
	Day 14	-0.26	0.53	-0.26	0.53	0.26	0.53	0.10	0.82	0.10	0.82	-0.10	0.82	0.10	0.82	-0.26	0.53	-0.07	0.87
NOD-*scid Il2rγ^null^ mice*	Day 1	-0.79	0.02	-0.79	0.02	0.79	0.02	0.88	0.00	0.64	0.09	-0.64	0.09	0.64	0.09	-0.79	0.02	0.74	0.04
	Day 14	-0.17	0.69	-0.17	0.69	0.17	0.69	0.48	0.23	0.48	0.23	-0.48	0.23	0.48	0.23	-0.17	0.69	0.79	0.02

Abbreviations: *1: serotransferrin, 2: beta-actin-like protein 2 , 3: serum albumin, 4: beta-2-glycoprotein 1, 5: inter-alpha-trypsin inhibitor heavy chain H2, 6: alpha-2-HS-glycoprotein, 7: hemoglobin subunit alpha, 8: hemoglobin subunit epsilon, 9: vitamin D-binding protein*

[#]When p-value <0.05, it was considered there were an association between protein corona and lung infiltration. rS-values close to -1 or +1 represent stronger relationships than values closer to zero.

Table S5. Correlation Analysis Between Protein Corona and AST

		1*		2		3		4		5		6		7		8		9	
		rS[#]	*P*	*rS*	*P*	*rS*	*P*	*rS*	*P*	*rS*	*P*	*rS*	*P*	*rS*	*P*	*rS*	*P*	*rS*	*P*
ICR/CD1 mice	Day 1	-0.48	0.233	-0.48	0.233	0.48	0.233	0.00	1.000	0.38	0.352	-0.38	0.352	0.38	0.352	-0.48	0.233	-0.10	0.823
	Day 14	-0.45	0.260	-0.45	0.260	0.45	0.260	0.31	0.456	0.45	0.260	-0.45	0.260	0.45	0.260	-0.45	0.260	0.31	0.456
NOD-scid *Il2rγ^null^* mice	Day 1	-0.86	0.007	-0.86	0.007	0.86	0.007	0.76	0.028	0.76	0.028	-0.76	0.028	0.76	0.028	-0.86	0.007	0.67	0.071
	Day 14	-0.26	0.528	-0.26	0.528	0.26	0.528	0.42	0.301	0.44	0.272	-0.44	0.272	0.44	0.272	-0.26	0.528	0.60	0.117

Abbreviations: *1: serotransferrin, 2: beta-actin-like protein 2 , 3: serum albumin, 4: beta-2-glycoprotein 1, 5: inter-alpha-trypsin inhibitor heavy chain H2, 6: alpha-2-HS-glycoprotein, 7: hemoglobin subunit alpha, 8: hemoglobin subunit epsilon, 9: vitamin D-binding protein*

[#]When p-value <0.05, it was considered there were an association between protein corona and AST. rS-values close to -1 or +1 represent stronger relationships than values closer to zero.

Table S6. Correlation Analysis Between Protein Corona and ALT

		1*		2		3		4		5		6		7		8		9	
		rS#	*P*	*rS*	*P*	*rS*	*P*	*rS*	*P*	*rS*	*P*	*rS*	*P*	*rS*	*P*	*rS*	*P*	*rS*	*P*
ICR/CD1 mice	Day 1	0.33	0.420	0.33	0.420	-0.33	0.420	-0.14	0.736	0.14	0.736	-0.14	0.736	0.14	0.736	0.33	0.420	0.33	0.420
	Day 14	0.05	0.911	0.05	0.911	-0.05	0.911	-0.17	0.693	-0.41	0.320	0.41	0.320	-0.41	0.320	0.05	0.911	-0.52	0.183
NOD-*scid* $Il2r\gamma^{null}$ mice	Day 1	-0.85	0.007	-0.85	0.007	0.85	0.007	0.64	0.091	0.90	0.002	-0.90	0.002	0.90	0.002	-0.85	0.007	0.86	0.062
	Day 14	-0.52	0.183	-0.52	0.183	0.52	0.183	0.83	0.010	0.50	0.207	-0.50	0.207	0.50	0.207	-0.52	0.183	0.81	0.015

Abbreviations: *1: serotransferrin, 2: beta-actin-like protein 2 , 3: serum albumin, 4: beta-2-glycoprotein 1, 5: inter-alpha-trypsin inhibitor heavy chain H2, 6: alpha-2-HS-glycoprotein, 7: hemoglobin subunit alpha, 8: hemoglobin subunit epsilon, 9: vitamin D-binding protein*

#When p-value <0.05, it was considered there were an association between protein corona and ALT. rS-values close to -1 or +1 represent stronger relationships than values closer to zero.

Table S7. Correlation of Protein Corona with Blood Cells in ICR/CD1 Mice

	1*		2		3		4		5		6		7		8		9	
	rS#	*P*	*rS*	*P*	*rS*	*P*	*rS*	*P*	*rS*	*P*	*rS*	*P*	*rS*	*P*	*rS*	*P*	*rS*	*P*
RBC	0.24	0.570	0.24	0.570	-0.24	0.570	-0.05	0.910	0.05	0.910	-0.05	0.910	-0.2	0.630	0.24	0.570	0.24	0.570
HGB	-0.29	0.490	-0.29	0.490	0.29	0.490	0.45	0.260	0.5	0.210	-0.5	0.210	0.5	0.210	-0.29	0.490	0.67	0.070
PLT	0.33	0.420	0.33	0.420	-0.33	0.420	-0.57	0.140	-0.57	0.140	0.57	0.140	-0.57	0.140	0.33	0.420	-0.81	0.015
HCT	0.12	0.780	0.12	0.780	-0.12	0.780	-0.07	0.870	0.07	0.870	-0.07	0.870	0.07	0.870	0.12	0.780	0.12	0.780
MCHC	-0.83	0.010	-0.83	0.010	0.83	0.010	0.79	0.021	0.74	0.037	-0.74	0.037	0.74	0.037	-0.83	0.010	0.69	0.060
PCV	-0.19	0.650	-0.19	0.650	0.19	0.650	0.34	0.410	0.43	0.280	-0.43	0.280	0.43	0.280	-0.19	0.650	0.58	0.130
MCH	-0.95	0.000	-0.95	0.000	0.95	0.000	0.69	0.060	0.64	0.090	-0.64	0.090	0.64	0.090	-0.95	0.000	0.38	0.350
RDW	-0.08	0.840	-0.08	0.840	0.08	0.840	-0.08	0.840	-0.11	0.800	0.11	0.800	-0.11	0.800	-0.08	0.840	-0.28	0.510
MCV	-0.66	0.070	-0.66	0.070	0.66	0.070	0.23	0.590	0.35	0.400	-0.35	0.400	0.35	0.400	-0.66	0.070	-0.08	0.840
WBC	0.67	0.070	0.67	0.070	-0.67	0.070	-0.67	0.070	-0.86	0.007	0.86	0.007	-0.86	0.007	0.67	0.070	-0.86	0.007
% Neutrophils	-0.45	0.260	-0.45	0.260	0.45	0.260	0.14	0.740	0.43	0.290	-0.43	0.290	0.43	0.290	-0.45	0.260	0.12	0.780
% Lymphocytes	-0.06	0.890	-0.06	0.890	0.06	0.890	0.34	0.380	-0.07	0.870	0.07	0.870	-0.07	0.870	-0.06	0.890	0.23	0.590
% Monocytes	-0.34	0.410	-0.34	0.410	0.34	0.410	0.24	0.560	0.15	0.730	-0.15	0.730	0.15	0.730	-0.34	0.410	0.05	0.910
% Eosinophils	0.24	0.580	0.24	0.580	-0.24	0.580	-0.54	0.160	-0.35	0.400	0.35	0.400	-0.35	0.400	0.24	0.580	-0.66	0.080

Abbreviations: *1: serotransferrin, 2: beta-actin-like protein 2 , 3: serum albumin, 4: beta-2-glycoprotein 1, 5: inter-alpha-trypsin inhibitor heavy chain H2, 6: alpha-2-HS-glycoprotein, 7: hemoglobin subunit alpha, 8: hemoglobin subunit epsilon, 9: vitamin D-binding protein*

#When p-value <0.05, it was considered there were an association between protein corona and blood cells. rS-values close to -1 or +1 represent stronger relationships than values closer to zero.

Table S8. Correlation of Protein Corona with Blood Cells in NOD-*scid* $Il2r\gamma^{null}$ Mice

	1*		2		3		4		5		6		7		8		9	
	rS#	*P*	*rS*	*P*	*rS*	*P*	*rS*	*P*	*rS*	*P*	*rS*	*P*	*rS*	*P*	*rS*	*P*	*rS*	*P*
RBC	-0.83	0.010	-0.83	0.010	0.83	0.010	0.79	0.021	0.74	0.037	-0.69	0.058	0.86	0.007	-0.83	0.010	0.69	0.060
HGB	-0.79	0.021	-0.79	0.021	0.79	0.021	0.83	0.010	0.69	0.060	-0.74	0.037	0.84	0.010	-0.79	0.021	0.74	0.037
PLT	0.67	0.070	0.67	0.070	-0.67	0.070	-0.86	0.007	-0.67	0.070	0.67	0.070	-0.67	0.070	0.67	0.070	-0.86	0.007
HCT	-0.83	0.010	-0.83	0.010	0.83	0.010	0.76	0.028	0.57	0.140	-0.57	0.140	0.57	0.140	-0.83	0.010	0.5	0.210
MCHC	-0.21	0.610	-0.21	0.610	0.21	0.610	0.48	0.230	0.48	0.230	-0.48	0.230	0.48	0.230	-0.21	0.610	0.74	0.037
PCV	-0.6	0.110	-0.6	0.110	0.6	0.110	0.16	0.190	0.35	0.400	-0.35	0.400	0.35	0.400	-0.6	0.110	0.27	0.530
MCH	-0.56	0.150	-0.56	0.150	0.56	0.150	0.28	0.510	0.68	0.060	-0.68	0.060	0.68	0.060	-0.56	0.150	0.4	0.330
RDW	0.42	0.300	0.42	0.300	-0.42	0.300	-0.17	0.690	-0.31	0.450	0.31	0.450	-0.31	0.450	0.42	0.300	-0.06	0.890
MCV	0.19	0.650	0.19	0.650	-0.19	0.650	-0.5	0.210	-0.45	0.260	0.45	0.260	-0.45	0.260	0.19	0.650	-0.76	0.030
WBC	-0.76	0.031	-0.76	0.031	0.76	0.031	0.83	0.011	0.71	0.050	-0.71	0.050	0.71	0.050	-0.76	0.031	0.78	0.023
% Neutrophils	-0.64	0.090	-0.64	0.090	0.64	0.090	0.69	0.060	0.83	0.010	-0.83	0.010	0.83	0.010	-0.64	0.090	0.88	0.004
%Lymphocytes	0.87	0.005	0.87	0.005	-0.87	0.005	-0.71	0.050	-0.83	0.011	0.83	0.011	-0.83	0.011	0.87	0.005	-0.66	0.080
% Monocytes	-0.12	0.780	-0.12	0.780	0.12	0.780	-0.07	0.870	0.07	0.870	-0.07	0.870	0.07	0.870	-0.12	0.780	-0.12	0.780
% Eosinophils	0.51	0.200	0.51	0.200	-0.51	0.200	-0.63	0.100	-0.53	0.180	0.53	0.180	-0.53	0.180	0.51	0.200	-0.65	0.080

***Abbreviations:** 1: serotransferrin, 2: beta-actin-like protein 2, 3: serum albumin, 4: beta-2-glycoprotein 1, 5: inter-alpha-trypsin inhibitor heavy chain H2, 6: alpha-2-HS-glycoprotein, 7: hemoglobin subunit alpha, 8: hemoglobin subunit epsilon, 9: vitamin D-binding protein*

#When p-value <0.05, it was considered there were an association between protein corona and blood cells. rS-values close to -1 or +1 represent stronger relationships than values closer to zero.

Table S9 Correlation of Lung injuries with RBC, HGB, HCT, MCHC, MCH and PCV That Reflecting Oxygen Transport Functions.

	ICR/CD1 mice Day 1	NOD-*scid* $Il2r\gamma^{null}$ mice Day 1
Kendall's concordance coefficient	0.981	0.974
*P-value**	0.000	0.000

**When P-value <0.05, Kendall's concordance coefficient is closer to 1 indicating strong association between lung injuries with oxygen transport functions.*

Table S10. Correlation Analysis Between Protein Corona and MCP-1

		1*		2		3		4		5		6		7		8		9	
		rS[#]	*P*	*rS*	*P*	*rS*	*P*	*rS*	*P*	*rS*	*P*	*rS*	*P*	*rS*	*P*	*rS*	*P*	*rS*	*P*
ICR/CD1 mice	Day 1	-0.19	0.65	-0.19	0.65	0.19	0.65	-0.07	0.87	0.26	0.53	-0.26	0.53	0.26	0.53	-0.19	0.65	0.00	1.00
	Day 14	0.93	0.000	0.93	0.000	-0.93	0.000	-0.83	0.010	-0.69	0.060	0.69	0.060	-0.69	0.060	0.93	0.000	-0.60	0.12
NOD-scid *Il2r*γ^{null} mice	Day 1	-0.17	0.69	-0.17	0.69	0.17	0.69	0.05	0.91	0.52	0.18	-0.52	0.18	0.52	0.18	-0.17	0.69	0.41	0.32
	Day 14	0.79	0.020	0.79	0.020	0.79	0.020	-0.48	0.230	-0.48	0.230	0.48	0.230	-0.48	0.230	0.79	0.020	-0.17	0.69

***Abbreviations:** 1: serotransferrin, 2: beta-actin-like protein 2 , 3: serum albumin, 4: beta-2-glycoprotein 1, 5: inter-alpha-trypsin inhibitor heavy chain H2, 6: alpha-2-HS-glycoprotein, 7: hemoglobin subunit alpha, 8: hemoglobin subunit epsilon, 9: vitamin D-binding protein.*

[#]When p-value <0.05, it was considered there were an association between protein corona and MCP-1. rS-values close to -1 or +1 represent stronger relationships than values closer to zero.

Table S11. Correlation Analysis Between Protein Corona and IL-6

		1*		2		3		4		5		6		7		8		9	
		rS[#]	*P*	*rS*	*P*	*rS*	*P*	*rS*	*P*	*rS*	*P*	*rS*	*P*	*rS*	*P*	*rS*	*P*	*rS*	*P*
ICR/CD1 mice	Day 1	0.33	0.42	0.33	0.42	-0.33	0.42	-0.14	0.74	0.14	0.74	-0.14	0.74	0.14	0.74	0.33	0.42	0.33	0.42
	Day 14	0.26	0.53	0.26	0.53	-0.26	0.53	-0.07	0.87	-0.31	0.46	0.31	0.44	-0.31	0.46	0.26	0.53	-0.12	0.78
NOD-scid *Il2r*γ^{null} mice	Day 1	-0.57	0.14	-0.57	0.14	0.57	0.14	0.52	0.18	0.62	0.10	-0.62	0.10	0.62	0.10	-0.57	0.14	0.57	0.14
	Day 14	0.26	0.53	0.26	0.53	-0.26	0.53	-0.17	0.69	-0.21	0.61	0.21	0.61	-0.21	0.61	0.26	0.53	-0.12	0.78

***Abbreviations:** 1: serotransferrin, 2: beta-actin-like protein 2 , 3: serum albumin, 4: beta-2-glycoprotein 1, 5: inter-alpha-trypsin inhibitor heavy chain H2, 6: alpha-2-HS-glycoprotein, 7: hemoglobin subunit alpha, 8: hemoglobin subunit epsilon, 9: vitamin D-binding protein*

. [#]When p-value <0.05, it was considered there were an association between protein corona and IL-6. rS-values close to -1 or +1 represent stronger relationships than values closer to zero.

Table S12. Correlation Analysis Between Protein Corona and TNF- α

		1*		2		3		4		5		6		7		8		9	
		rS[#]	*P*	*rS*	*P*	*rS*	*P*	*rS*	*P*	*rS*	*P*	*rS*	*P*	*rS*	*P*	*rS*	*P*	*rS*	*P*
ICR/CD1 mice	Day 1	-0.14	0.74	-0.14	0.74	0.14	0.74	0.52	0.18	0.24	0.57	-0.24	0.57	0.24	0.57	-0.14	0.74	0.62	0.10
	Day 14	0.50	0.21	0.50	0.21	-0.50	0.21	-0.17	0.70	-0.60	0.12	0.60	0.12	-0.60	0.12	0.50	0.21	-0.26	0.53
NOD-scid $Il2r\gamma^{null}$ mice	Day 1	0.53	0.18	0.53	0.18	-0.53	0.18	-0.44	0.27	-0.23	0.59	0.23	0.59	-0.23	0.59	0.53	0.18	-0.14	0.73
	Day 14	-0.17	0.69	-0.17	0.69	0.17	0.69	-0.02	0.96	0.41	0.32	-0.40	0.32	0.41	0.32	-0.17	0.69	0.21	0.61

***Abbreviations:** 1: serotransferrin, 2: beta-actin-like protein 2 , 3: serum albumin, 4: beta-2-glycoprotein 1, 5: inter-alpha-trypsin inhibitor heavy chain H2, 6: alpha-2-HS-glycoprotein, 7: hemoglobin subunit alpha, 8: hemoglobin subunit epsilon, 9: vitamin D-binding protein.*

[#]When p-value <0.05, it was considered there were an association between protein corona and TNF- α. rS-values close to -1 or +1 represent stronger relationships than values closer to zero.

Table S13. Correlation of Cytokines and Chemokines with Lung and Liver Injuries in ICR/CD1 and NOD-*scid* *Il2rγ*^{null} Mice

	Relative Lung Infiltration (%)				AST and ALT			
	ICR/CD1 mice		NOD-*scid Il2rγ*^{null} mice		ICR/CD1 mice		NOD-*scid Il2rγ*^{null} mice	
	Day 1	Day 14	Day 1	Day 14	Day 1	Day 14	Day 1	Day 14
Kendall's concordance coefficient	**0.906**	**0.925**	**0.906**	**0.925**	**0.953**	**1.000**	**0.906**	**0.963**
*P-value**	0.000	0.000	0.000	0.000	0.000	0.000	0.000	0.000

**When P-value <0.05, Kendall's concordance coefficient is closer to 1 indicating strong association between cytokines with lung injury or liver injury.*

Table S14. Correlation of WBC with Lung and Liver Injuries in ICR/CD1 and NOD-*scid* *Il2rγ*^{null} Mice

	% lung relative infiltration		AST and ALT	
	ICR/CD1 mice	NOD-*scid Il2rγ*^{null} mice	ICR/CD1 mice	NOD-*scid Il2rγ*^{null} mice
	1-day	1-day	1-day	1-day
Kendall's concordance coefficient	0.931	0.903	0.946	0.945
P-value	0.000	0.000	0.000	0.000

** When P-value <0.05, Kendall's concordance coefficient is closer to 1 indicating strong association between WBC with lung injury or liver injury.*

Table S15. Correlation of Cytokines and Chemokines with WBC

	WBC	
	ICR/CD1 mice<br>Day 1	NOD-*scid Il2rγ*^{null} mice<br>Day 1
Kendall's concordance coefficient	**0.844**	**0.881**
*P-value**	0.000	0.000

**When P-value <0.05, Kendall's concordance coefficient is closer to 1 indicating strong association between cytokines with WBC.*
